# Fossilization transforms vertebrate hard tissue proteins into N-heterocyclic polymers

**DOI:** 10.1038/s41467-018-07013-3

**Published:** 2018-11-09

**Authors:** Jasmina Wiemann, Matteo Fabbri, Tzu-Ruei Yang, Koen Stein, P. Martin Sander, Mark A. Norell, Derek E. G. Briggs

**Affiliations:** 1 Department of Geology & Geophysics, Yale University, 210 Whitney Avenue, New Haven, CT 06511 USA; 2 Steinmann Institute for Geology, Mineralogy, and Paleontology, University of Bonn, Nussallee 8, 53115 Bonn, Germany; 3 Earth System Sciences AMGC, Vrije Universiteit Brussel, Pleinlaan 2, 1050 Brussels, Belgium; 4 Dinosaur Institute, Natural History Museum of Los Angeles County, 900 Exposition Boulevard, Los Angeles CA, 90007 USA; 5 Division of Vertebrate Paleontology, American Museum of Natural History, Central Park West at 79th Street, New York, NY 10024-5192 USA

## Abstract

Vertebrate hard tissues consist of mineral crystallites within a proteinaceous scaffold that normally degrades post-mortem. Here we show, however, that decalcification of Mesozoic hard tissues preserved in oxidative settings releases brownish stained extracellular matrix, cells, blood vessels, and nerve projections. Raman Microspectroscopy shows that these fossil soft tissues are a product of diagenetic transformation to Advanced Glycoxidation and Lipoxidation End Products, a class of N-heterocyclic polymers generated via oxidative crosslinking of proteinaceous scaffolds. Hard tissues in reducing environments, in contrast, lack soft tissue preservation. Comparison of fossil soft tissues with modern and experimentally matured samples reveals how proteinaceous tissues undergo diagenesis and explains biases in their preservation in the rock record. This provides a target, focused on oxidative depositional environments, for finding cellular-to-subcellular soft tissue morphology in fossils and validates its use in phylogenetic and other evolutionary studies.

## Abstract (web summary)

Recent studies have reported preservation of proteinaceous soft tissues within dinosaur bones. Here, Wiemann et al. combine analyses of fossil vertebrate tissues and experimentally matured modern samples to elucidate the mechanism of soft tissue preservation and the environments that favor it.

## Introduction

Proteins determine organismic form and function. They constitute extracellular matrices, cells, blood vessels, and nerve projections which themselves assemble higher-level tissue architectures. Such soft tissues are decay prone and their preservation requires particular conditions that inhibit decay and promote stabilization through diagenesis^1–3^. When such proteinaceous soft tissues are fossilized, they provide remarkable insights into the nature and biology of extinct animals^4–7^. Data from ancient proteinaceous soft tissues have been used for phylogenetic analysis^8,9^, ecological inferences^7,8^, and for reconstructing physiology^10,11^. Nonetheless their preservation in deep time is still regarded as controversial^12–15^. Vertebrate hard tissues represent a model system for understanding protein diagenesis based on the antagonistic effects of mineral stabilization and protein hydrolysis^13^. The maximum longevity of original proteinaceous matter in vertebrate hard tissues has been estimated at 3.8 million years, although molecular remnants have been reported from older rocks^13,14,16,17^. Thus, the preservation of originally proteinaceous soft tissues in Mesozoic fossils, although independently confirmed for oligopeptide-grade degradation products^18–20^, appears anomalous not least because the preservation of originally proteinaceous remnants in fossil vertebrate hard tissues seems to be biased towards oxidative depositional environments^18,21^, which are thought to favor decay. Reconciling this apparent contradiction requires a general mechanism to explain the potential transformation and stabilization of proteinaceous matter through diagenesis over millions of years^22^. Such preservation has been attributed to isolation and stabilization by incorporation into minerals^23–25^, organo-metallic complexing^26^, and physical or chemical binding to mineral surfaces^13,27^, and anhydrous sugar-protein crosslinking processes^28,29^, but none of these models provides an explanation for patterns of originally proteinaceous soft tissue preservation in vertebrate hard tissues in deep time. This may be due in part to a reliance on analytical methods, such as liquid chromatography, mass spectrometry, immunological techniques, and protein sequencing, which target a particular molecular structure and make it difficult to address the general question of how soft tissues fossilize. Alternative considerations posit that Mesozoic soft tissues are not original, but represent replication of vascular canals and cell lacunae by more recent microbial biofilms^12^.

Glycoxidation and lipoxidation of proteins in vivo results in medical conditions related to aging. In food chemistry, they transform proteins to colorants and yield aromas^30^. Advanced Glycoxidation Endproducts (AGEs) and Advanced Lipoxidation Endproducts (ALEs) are N-heterocyclic polymers characterized by brown-stained, crosslinked amino acid residues^30,31^. AGEs and ALEs can accumulate in reactant-enriched substrates due to thermal maturation, especially when their formation is promoted by oxygen, water, phosphates, and slightly alkaline conditions, or catalyzed by transition metals (e.g., iron)^30^. Such oxidative changes affect all structures with a proteinaceous scaffold. Collagen, for example, which is the most abundant structural protein in vertebrates, loses its elasticity and becomes brittle^32^; crosslinking thickens the collagenous vascular wall of blood vessels^33^. AGEs and ALEs resist microbial digestion by knocking out catalytic, active sites of proteolytic enzymes^30,34^. All such processes increase the fossilization potential of proteinaceous soft tissues.

We use Raman Microspectroscopy, the favored method for AGE and ALE detection in biomedicine and food chemistry^35,36^, to investigate the composition of soft tissues within a diversity of fossil vertebrate hard tissues, and to test whether their preservation is related to oxidative burial conditions. Raman spectroscopy has the advantage that it provides a general picture of all the inorganic and organic compounds within a sample. The color and composition of fossil host rocks provide a proxy for burial conditions^37^. Sediment color is strongly influenced by Eh, which determines the ratio of Fe^2+^ to Fe^2+^ + Fe^3+^, and by the concentration of host rock organics. Our samples are from silicate and carbonate sedimentary rocks which lack staining minerals other than iron oxides; clay minerals and pyrite, which also yield darker (gray/black) colors, are only present in a small proportion of our fossil host rocks (Supplementary Tables 1–5). Thus red, purple (high Eh), and green/olive gray sediment colors (medium Eh) indicate oxidative conditions, in contrast to gray to black sedimentary rocks which indicate reducing conditions (low Eh)^37^. These inferences are also based on previous case studies of the strata of interest (Supplementary Table 5). We also compare molecular signatures generated by experimental maturation of modern vertebrate hard tissues to those in fossils. These approaches demonstrate that only specimens from oxidative depositional environments preserve chemically transformed soft tissues, allowing us to infer that proteinaceous matter transforms into AGEs/ALEs, a class of N-heterocyclic polymers, during fossilization. Based on molecular and statistical data, we predict that oxidative depositional environments are likely to yield morphological preservation of soft tissues in fossils.

## Results

### Soft tissues preserve in oxidative settings

Twenty-four specimens (Supplementary Tables 1–5, Supplementary Figs. 1–3) of biomineralized vertebrate tissues ranging in age from modern to Late Triassic (ca. 205 mya), and representing environments from oxidative to reducing, were decalcified to release any soft tissues present, and their mineralogy and organic content were analyzed (Supplementary Table 1–5). All specimens exhibited dark brown to gray-black colors (Supplementary Figs. 1–2). Decalcification did not affect key molecular features and their potential transformation products in soft tissues (Supplementary Fig. 4).

**Fig. 1:**
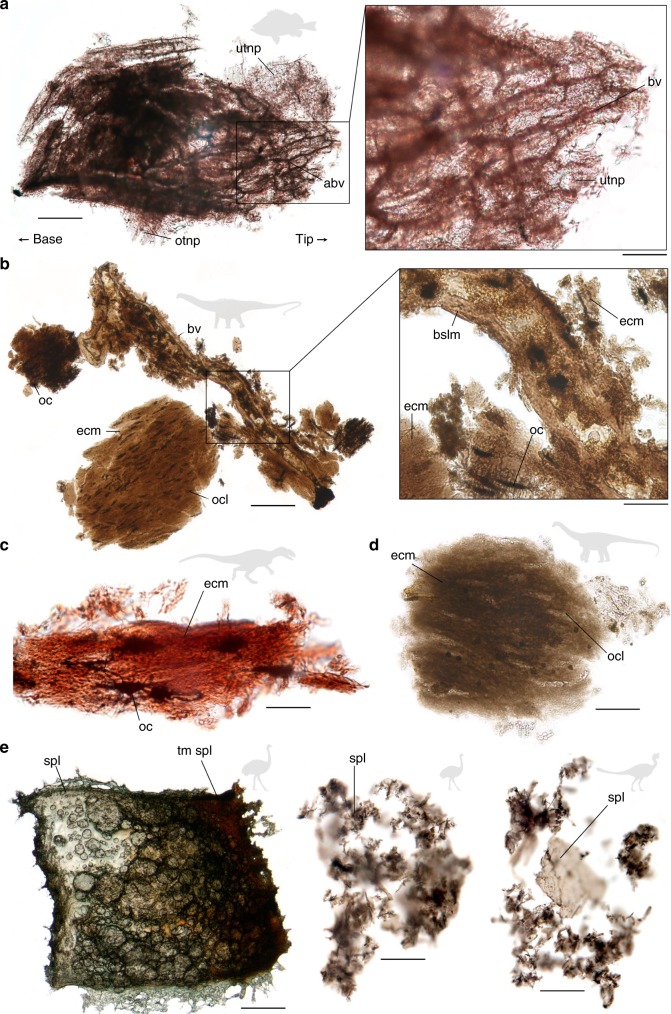
Decalcified vertebrate hard tissues (representing a total of 7 specimens). **a** Paleonisciform ganoid scale (Oxfordian (Jurassic), Xinjiang, China) showing articulated blood vessels (abv) of the dentine and organic matrix with peripheral aligned and ordered (otpn), or unordered (utnp), tubular nerve projections. The left scale bar equals 500 μm, the right one 250 μm. **b** Blood vessel (bv) fragments from a diplodocid sauropod (Jurassic, Wyoming, US). The basal lamina (bslm) shows original stratification even though the vascular wall is thickened compared to modern archosaur blood vessels. Osteocytes (oc) with dense filipodia (f) are embedded in the originally collagenous extracellular matrix (ecm). Osteocytes adjacent to the blood vessel preserve cellular detail, but elsewhere they have degraded leaving the osteocyte lacunae (ocl) in the organic matrix. The left scale bar equals 100 μm, the right one 25 μm. **c** Extracellular matrix from an *Allosaurus fragilis* vertebra (Late Jurassic, Wyoming, US). The originally collagenous matrix fibers are preserved, and osteocytes with filipodia are dark and infilled. The scale bar equals 25 μm. **d** Extracellular matrix from an *Apatosaurus* sp. bone (Late Jurassic). Osteocyte lacunae are preserved. Decalcified avian and non-avian dinosaur eggshells. The scale bar equals 25 μm. **e** (Left) *Rhea* (modern, in captivity, Montana, US), artificially matured spongy layer. The maturation gradient increases to the right of the image. The original spongy layer is green due to pigmentation, while the matured part is brown. The scale bar equals 500 μm. (Center) *Psammornis rothschildi* (Holocene, Algeria), degraded spongy layer fragments. The scale bar equals 25 μm. (Right) Oviraptorid *Heyuannia huangi* (Late Cretaceous, Jiangxi, China) spongy layer. The scale bar equals 25 μm

Soft tissues were present in all modern samples, but only in those fossils from oxidative settings. The Jurassic (Oxfordian) paleonisciform scales preserve a fully articulated three-dimensional vascular system together with a dense meshwork of unbranched tubular nerve projections resembling the dental tubuli in modern vertebrate teeth (Fig. 1a, Supplementary Fig. 1), the first such features discovered in a fossil. The blood vessels are stained brown. The tubular nerve projections are hollow and appear translucent with walls that are beige in color. The soft tissues are brittle and cracked, and account for about 70% by volume of the ganoid scale prior to decalcification (compare Fig. 1a, Supplementary Fig. 1).

The organic remains in the bone samples are also brittle. The Jurassic diplodocid bone yielded large (up to 1500 µm) extracellular matrix fragments which preserve anastomosing vascular canals and interconnected (max. 25 µm length) osteocytes with extensive filipodia in some areas, but only osteocyte lacunae elsewhere (Fig. 1b, compare Fig. 1d). The extracellular matrix harboring the osteocytes is the most intensively stained, and occurs as homogenous patches without any identifiable fibers. The Jurassic *Allosaurus fragilis* preserves an extracellular matrix made up of fibers resembling collagen (Fig. 1c). Osteocytes (max. 25 µm length) are evident as three-dimensional opaque ellipsoidal structures with associated filipodia. The Jurassic *Apatosaurus* sp. preserves only small patches of extracellular matrix (up to 150 µm in dimension) comprising a homogenous organic scaffold (Fig. 1d). Neither osteocytes nor blood vessels are preserved, but ellipsoidal osteocyte lacunae are evident as impressions in the organic matrix.

Experimentally matured (see Methods Experimental Maturation) modern *Rhea americana* (Fig. 1e) eggshell yielded a spongy layer with an intense brown color which disintegrated into small, sheath-like fragments (<30 µm). The eggshell of Holocene *Psammornis rothschildi* preserves similar fragments (up to 10 µm) stained blackish-brown (Fig. 1e). That of Cretaceous *Heyuannia huangi* preserves fragments up to 40 µm in length (Fig. 1e). The organic material in the fossil eggshells has lost its elasticity.

**Fig. 2:**
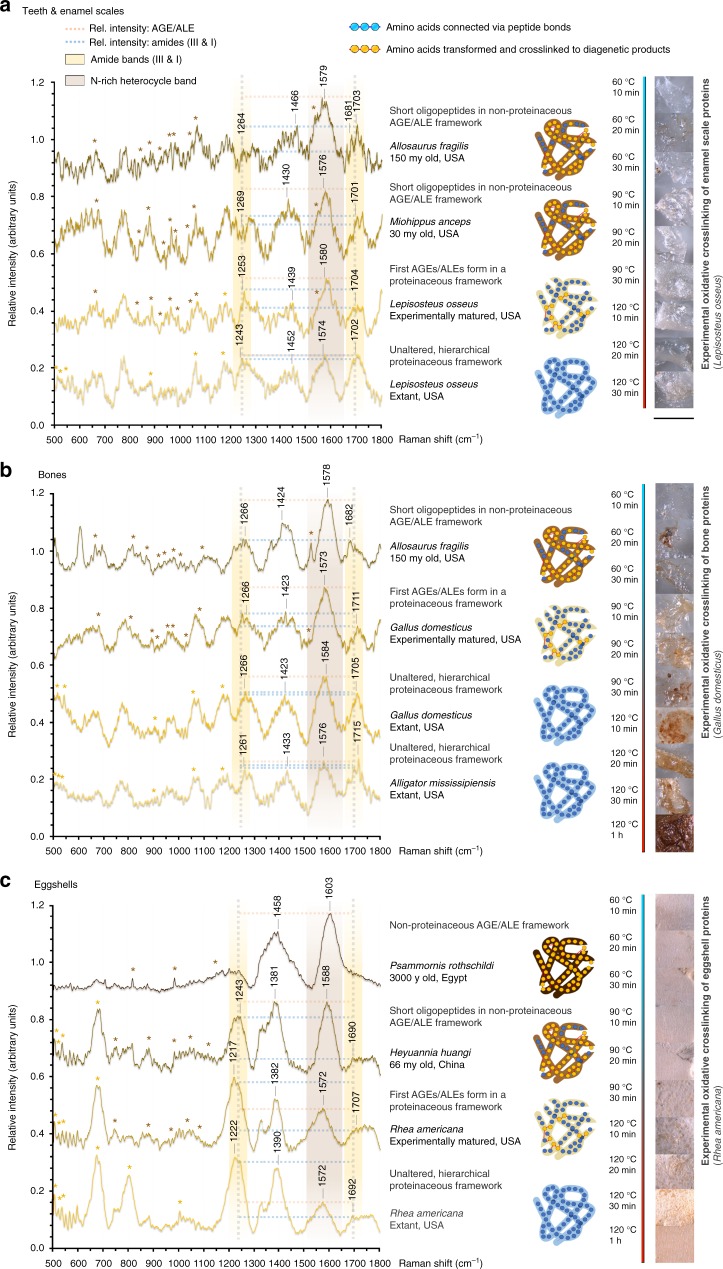
Aligned Raman spectra of decalcified modern, matured, and fossil organic material. All spectra were obtained in aqueous solution, 500–1800 cm^−1^, 20 s exposure time, 4 accumulations. These spectra are based on a total of 12 specimens with soft tissue preservation used for high-resolution point measurements, while Raman spectra for an additional 12 specimens without soft tissue preservation can be found in Supplementary Fig. 9; a total of additional 29 specimens was used for experimental maturation. **a** Teeth and enamel scales. **b** Bones. **c** Eggshells. Differences in the color of the sampled material are represented in the icons on the right. Brown spectral band: advanced glycoxidation and lipoxidation end products (AGEs), oxidative crosslinks; yellow spectral bands: peptide amides. AGE bands increase in intensity relative to amide I bands (dotted lines) with age and artificial maturation. Protein degradation and deamidation over time are represented by a decrease in the band intensities identified as amide III and amide I. Details on experimentally matured reference tissues at different temperatures (autoxidation) on the right of the spectra show that oxidative, brown discoloration already occurs at low temperatures. The color scale associated with temperatures and incubation times for the maturation experiments range from red (relatively high temperatures) to blue (low temperatures). The scale bar equals 500 μm

Experimental maturation (oxidative crosslinking) of modern soft tissues released by decalcification of enamel scales of *Lepisosteus osseus*, bones of *Gallus domesticus*, and eggshells of *Rhea americana*, resulted in discoloration from translucent white to progressively darker brown as temperature (45–120 °C) and duration of air exposure (10–60 min) were increased, yielding colors similar to those in the fossil soft tissues (compare Fig. 1, Fig. 2). Samples from reducing depositional environments did not release any soft tissue structures (Supplementary Fig. 9).

### Fossil soft tissues consist mainly of non-proteinaceous N-heterocyclic polymers

Raman Spectroscopy (Fig. 2) of extracted soft tissues yielded amide signals representing peptide bonds (Supplementary Table 3): amide III between 1230 and 1250 cm^−1^ and amide I between 1650 and 1690 cm^−1^ (Supplementary Table 6). AGE/ALE bands between 1550 and 1600 cm^−1^ (Supplementary Table 6) generally increase in intensity with the age of the fossil, whether enamel scale, tooth, bone, or eggshell (Fig. 2). This, the most prominent band in the spectra, is assigned to the C=C stretching of a (transition metal chelating) imidazole ring (vibrations at 1434–1440 cm^−1^ and 1340–1352 cm^−1^ confirm this assignment, Supplementary Table 6). In the modern samples, this band is far less prominent, and corresponds to the N-heterocycle of the amino acid tryptophane, and the few age-related AGEs/ALEs present (*Gallus* versus *Alligator* bone in Fig. 2). The spectra also show a N-heterocycle pyridine-like ring stretch component, minor carbonyl stretching at a higher wavenumber, and N-H deformation at lower wavenumbers, all characteristic of AGEs and ALEs^36,38^ (Fig. 2, Supplementary Table 6). The intensity of the AGE/ALE bands shows a reciprocal relationship to that of the amide bands^36,39^ (Supplementary Fig. 5).

### Oxidative crosslinking transforms proteins during fossilization

**Fig. 3:**
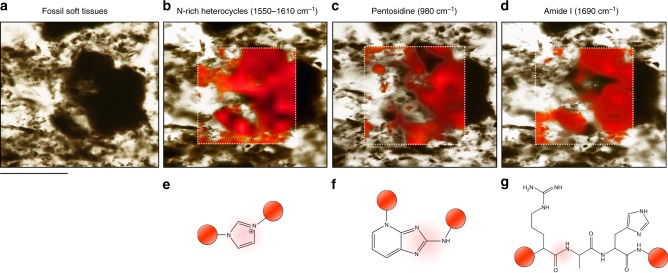
High-resolution Raman maps (3 s exposure time, 3 accumulations) of remains of extracellular matrix extracted from *Allosaurus fragilis* bone in aqueous hydrochloric acid (pH = 3). The dotted rectangles mark the area mapped, and the different shades of red represent the signal intensity of the protein fossilization product (PFP). The colored spheres framing the compound structures represent a N-heterocycle polymer context, and the red shading labels the functional unit that gives rise to each signal mapped out. The scale bar equals 300 μm. **a** Dark residue of soft tissue. **b** Map of N-rich heterocycles (1550–1610 cm^−1^), which cover most of the sample surface. **c** Pentosidine (980 cm^−1^), the AGE marker, which corresponds to the spatial distribution of N-rich heterocycles. **d** Amide I generated by peptide bonds, which is a less precise match for the N-rich heterocycles (1690 cm^−1^). **e**–**g** The organic functional units representing the selected Raman shifts are shaded (red) below each map

The ratio of AGE/ALE band intensity to amide I band intensity represents the proportion of oxidative crosslinks relative to unaltered protein/peptide mass^36,38,39^, and is reflected in the discoloration of extracted soft tissues (Fig. 2, Supplementary Table 4): An increased amount of AGEs/ALEs relative to unaltered peptides yields a more intense brown stain (Fig. 2, Supplementary Table 4). The AGE/ALE to amide I band intensity ratio of the experimentally matured modern soft tissue samples, and the intensity of their brown stain, fall between those of the unaltered modern samples and the fossil samples (Fig. 2, Supplementary Fig. 5). The Raman shift of the fossil soft tissue amide III and amide I bands is indicative of the structural organization of preserved crosslinked peptides, and indicates the presence of (unordered) secondary structures in most samples (Supplementary Table 6). Disulfide-bridges indicating the presence of tertiary structures are generally absent in fossil materials, showing that potentially preserved oligopeptides are of very short chain length (Fig. 2); some fossil samples appear to be composed of fully transformed, non-proteinaceous AGEs/ALEs (Fig. 2). This is corroborated in our Raman maps (Fig. 3): N-rich heterocycles produce a spatial signal restricted to soft tissue surfaces where they appear to be the major constituents (Fig. 3a, b). Pentosidine, a classic AGE marker, corresponds closely in its spatial distribution to the N-rich heterocyclic polymers (Fig. 3c), while the amide I signal (indicative of peptide bonds) is most prominent in areas with low N-rich heterocycle signal (Fig. 3d). High-resolution point spectra also revealed a range of minor compounds identified as degradation products of lipids (Fig. 2, Supplementary Table 6, Supplementary Fig. 6). In situ Raman spectra of vertebrate hard tissues from reducing environments showed no evidence for amide bands representing peptide bonds (Supplementary Fig. 9).

**Fig. 4:**
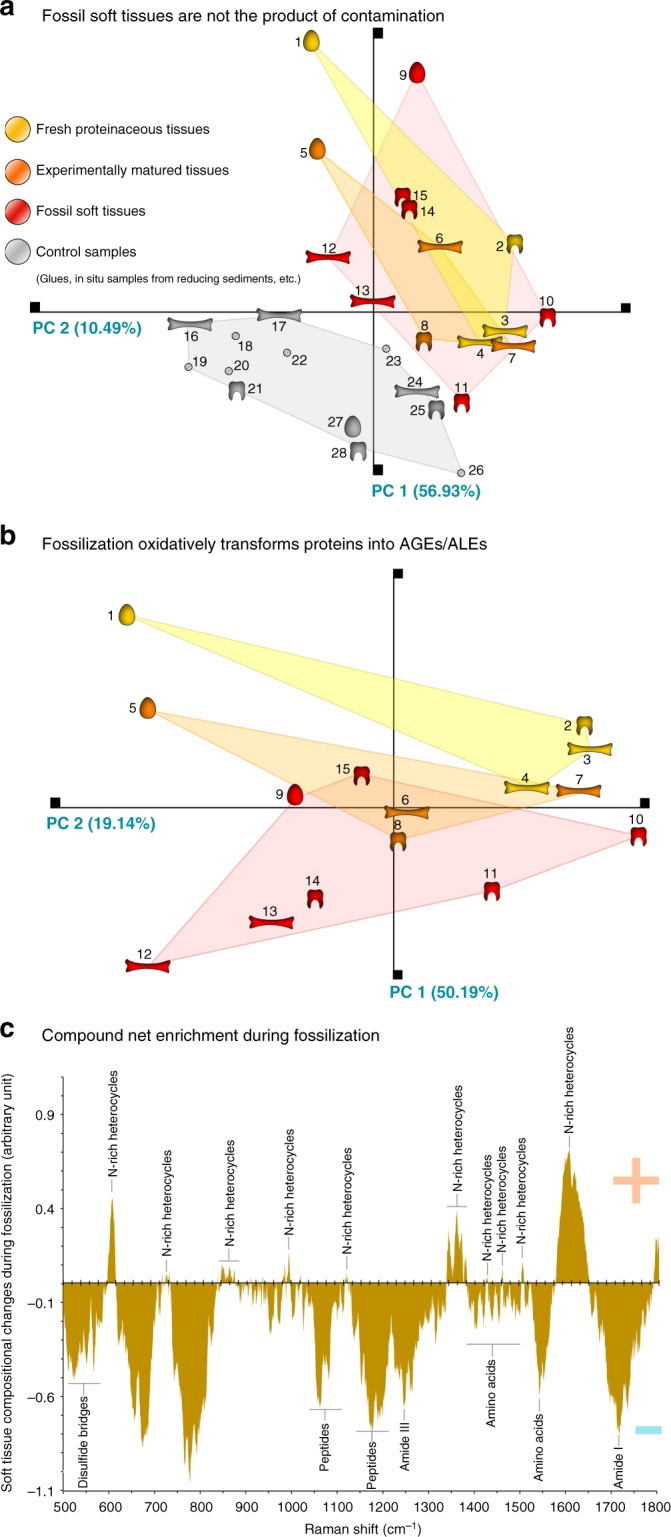
Chemospace and dissimilarity plot showing chemical similarity between modern, matured and fossil samples. **a** Chemospace including control samples (sediments, undecalcified samples from reducing settings, glues, epoxy resin; a total of 28 specimens). **b** Chemospace excluding control samples (a total of 15 specimens). Colored clusters represent the three main categories of samples analyzed in this study: control samples (gray), modern (yellow), experimentally matured (orange) and fossil hard tissues (red). 1 *Rhea americana* eggshell; 2 *Lepisosteus osseus* scale; 3 *Gallus domesticus* eggshell; 4 *Alligator mississipiensis* bone; 5 *Rhea americana* eggshell matured; 6 *Gallus domesticus* matured; 7 *Alligator mississipiensis* bone matured; 8 *Lepisosteus osseus* scale matured; 9 *Heyuannia huangi* eggshell; 10 *Miohippus anceps* tooth; 11 *Allosaurus fragilis* tooth; 12 *Psammornis rothschildi* eggshell; 13 *Allosaurus fragilis* bone; 14 paleonisciform scale; 15 paleonisciform scale; 16 crocodilian bone; 17 *Iguanodon bernissartensis* bone; 18 sediment surrounding *Diplodocus* bone; 19 epoxy resin; 20 sediment surrounding *Maiasaura* sp. remains; 21 *Maiasaura* sp. tooth; 22 sediment embedding *Ichthyosaurus* bone; 23 polyacrylamide glue; 24 *Ichthyosaurus* sp. bone; 25 *Maiasaura* sp. eggshell; 26 polyacrylamide glue on fossil bone; 27 *Saltasaurus* sp. eggshell; 28 *Nothosaurus* sp. tooth. **c** Dissimilarity plot illustrating soft tissue compositional changes through fossilization. An average Raman spectrum based on all fresh soft tissues (a total of 4 specimens acquired with 4 accumulations) was subtracted from that based on all fossil soft tissues analyzed (a total of 7 specimens acquired with 4 accumulations). The spectral bands indicating the net enrichment of proteinaceous soft tissues during fossilization correspond to pentosidine (depicted), a classic AGE/ALE marker

A Principal Component Analysis (PCA) based on spectral intensities at seventeen representative Raman shifts (compositionally informative on peptide bonds and N-heterocycles) showed for the complete set of modern, matured, fossil and control samples (including in situ spectra of samples from reducing depositional environments, and various potential contaminants) a clear separation between two clusters formed by control samples and soft tissues (Fig. 4a). An additional PCA (Fig. 4b) focusing on the signal within the spectra of fresh, matured, and fossil soft tissues revealed a cluster of fresh soft tissues which is distinct from the cluster of fossil soft tissues, and a third cluster of experimentally matured soft tissues that bridged the other two sample clusters. Comparing the average composition (*n* = 4) of fresh soft tissue with that of fossil soft tissue (*n* = 7) (Fig. 4c), reveals a net enrichment during fossilization that corresponds to the N-heterocyclic crosslink pentosidine. Pentosidine is a classic and reliable marker for AGEs and ALEs^30^.

## Discussion

The overlap of clusters of fossil and experimentally matured soft tissues in the PCA Chemospace (Fig. 4b) indicates that our experimentally crosslinked samples yield molecular signatures similar to those obtained from fossil soft tissues. AGE/ALE-corresponding N-rich heterocycles are identified as the net enrichment in tissue composition resulting from fossilization processes (Fig. 4c). These compounds were mapped out in extracted fossil soft tissues, corroborating the specificity of our Raman signals (Fig. 3). This combined evidence (Figs. 3, 4b, c) illustrates that the N-heterocycle polymer composition of the fossil samples is the result of diagenetic alteration of originally proteinaceous material; their chemistry is not consistent with contamination from modern organisms (Fig. 4a). Further support for this process of protein transformation into N-heterocyclic polymers of AGE/ALE type is offered by the characteristic brown stain^30^ of extracted fossil soft tissues (Fig. 1), and the bias in their preservation towards oxidative settings (Supplementary Tables 1–5). Thus, our investigation confirms that original morphological data can be obtained from soft tissue within fossil vertebrate hard tissues, and validates their use to address evolutionary questions.

AGEs and ALEs are generated via externally induced or oxoradical-mediated protein crosslinking^30^. The latter occurs in animals in vivo, with increased rates in endothermic taxa and those with high metabolic rates^30^. Our suite of modern samples represents a range of baseline AGE/ALE concentrations that correlate with metabolic rates from low in alligators to high in modern birds^40,41^ (Fig. 2). All our fossil samples exceed these baseline concentrations (Fig. 2). This diagenetic increase in AGEs/ALEs correlates with oxidative conditions as evidenced by the nature of the depositional environments in which they occur (Supplementary Tables 1–5), and by the generation of similar molecular and morphological signatures in artificially matured soft tissues (Figs. 2, 4; Supplementary Fig. 5), an approach applied in investigations of other fossil molecular material^42–44^. Oxidative crosslinks are already present in *Psammornis rothschildi* (3 ky, Fig. 2), and were prominent after experimental maturation at 60 °C for 10 min, suggesting that oxidative crosslinking is an early diagenetic process, dependent on chemical conditions in the depositional environment as well as later diagenetic processes, rather than increased temperatures due to burial.

**Fig. 5:**
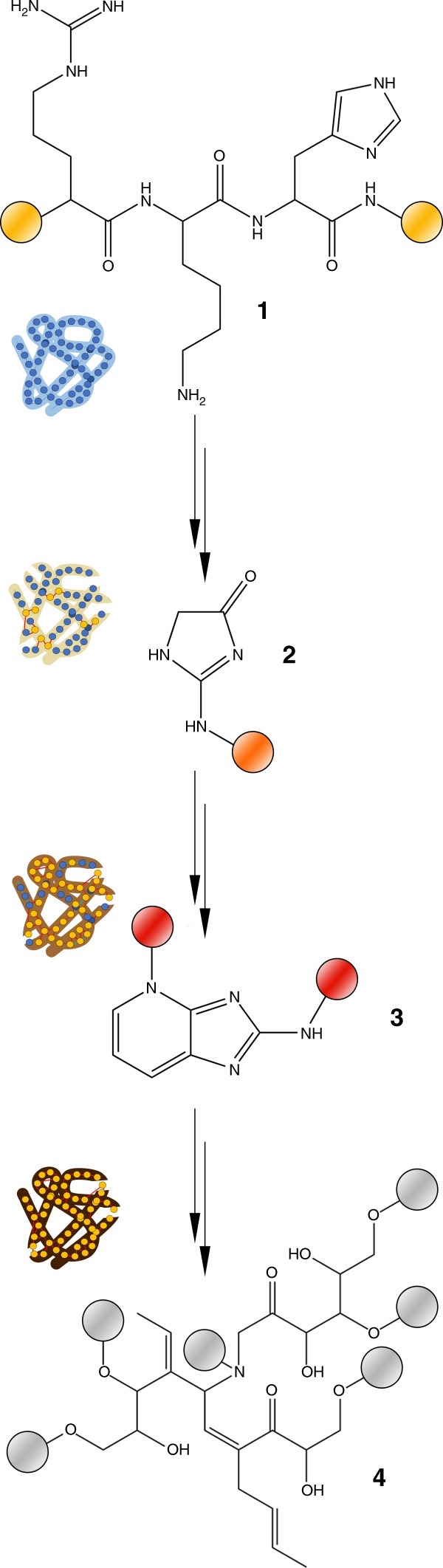
Schematic of the proposed transformations of proteins during fossilization in hard tissues. 1 Unaltered tripeptide containing arginine, lysine, and histidine residues. Peptides constitute proteins in fresh tissues. 2 Imidazolone is an early, non-crosslinking product of glycoxidation/lipoxidation reactions and can be found in altered proteinaceous matter of experimentally matured and fossil samples (Fig. 2). 3 A pentosidine crosslink, commonly found in advanced glycoxidation/lipoxidation end products based on originally proteinaceous matter, and in experimentally matured, and fossil samples (Fig. 2). 4 A highly oxidized, polymeric degradation product similar to a melanoidine. Structure may vary. Similar compounds were found in *Psammornis* (Fig. 2). The colored spheres represent a proteinaceous context (yellow), an oxidatively altered proteinaceous context (orange), a non-proteinaceous N-rich heterocyclic polymer as product of protein transformation (red), and a non-proteinaceous, peroxidized, polymeric scaffold (gray). The protein icons next to the structures illustrate the corresponding degree of tissue discoloration caused by enrichment in each compound as in Fig. 2

Blood vessels are connected to osteocytes via networks of filipodia and nerve projections, and provide a conduit for pore water, as well as reactive organic surfaces (Fig. 1). Iron ions mobilized by surface dissolution processes of surrounding sediments catalyze the formation of reactive oxygen species which contribute to oxidative alterations^30^. Alkaline conditions generated by the release of phosphate ions during hard tissue mineral dissolution promote AGE/ALE formation^30^, and gradual temperature increase during burial accelerates crosslink propagation (Fig. 5). This scenario is consistent with the spatial correspondence between N-rich heterocycles and the AGE marker pentosidine in fossil soft tissues, while amide signals indicative of intact peptide bonds occur mainly in areas that show low signal intensities for N-rich heterocycles (Fig. 3).

The generation of brown-stained proteinaceous material, and subsequently non-proteinaceous AGEs and ALEs, provides an explanation for the apparent anomaly of widespread morphological and molecular preservation of soft tissues in fossil vertebrate hard tissues. Both AGEs and ALEs exhibit hydrophobic behavior^30^ due to the chemical character of their crosslinks, which in turn shield adjacent peptides from hydrolysis^45^. Thermo-oxidatively induced, intensive crosslinking of proteins results in hydrophobic, reinforced AGE/ALE scaffolds resistant to microbial digestion. This explains the preservation of fragile soft tissues in certain chemical environments through deep time^30,39^.

The soft tissues we analyzed are a product of the transformation of endogenous proteins in oxidative terrestrial, lacustrine, and shallow marine settings. The absence of soft tissue residues and amide signals indicative of peptide bonds in dark sediments suggests that chemically reducing conditions, such as (non-bioturbated) deep marine deposits, are unlikely to yield soft tissue structures of comparable quality. In identifying brown vertebrate hard tissue fossils in light colored (oxidative) sediments as a target, our observation provides a first field guide to the search for endogenous soft tissues in fossil vertebrate remains as a basis for addressing a range of evolutionary questions.

## Methods

### Specimen samples

We investigated a total of 35 specimens (Figs. 2, 4, Supplementary Tables 1–4, Supplementary Figs. 1, 2, 3, 8, 9)—teeth and enamel scales (*n* = 8), bones (*n* = 10), and eggshells (*n* = 6)—from reducing (*n* = 9) and oxidative (*n* = 8) depositional environments^37^, as well as glue (*n* = 2), epoxy resin (*n* = 1), and sediment samples (*n* = 8).

### Specimen photography and microscopy

All specimens (Fig. 2, Supplementary Tables 1–4, Supplementary Figs. 1, 2) and available sediment samples (Supplementary Figs. 3) were cleaned with ethanol and photographed using a Leica MZ16 dissecting microscope with Optronics camera attachment. Samples were photographed prior to treatment. Leica Application Suite Core software was used for photography with a white balance presetting. Rock matrices of fossil hard tissues were categorized as reducing or oxidizing^37^ based on their color, mineral content, and previous studies (Supplementary Tab. 5, Supplementary Figs. 3, 8).

### Mineralogical assessment

A sample of each hard tissue specimen was pulverized and 3 g aliquots were analyzed using a Siemens D5000 powder diffractometer (Steinmann Institute, University of Bonn, Germany) operating at 45 kV and 40 A under CuKα radiation (wavelength 1.54 Å), variable divergence apertures, and a graphite secondary monochromator. The diffraction diagrams were recorded with an acquisition time of 4 s per step at 2*θ* = 0.02°. Mineral assignments were confirmed by Raman spectroscopic analyses of the sediments (Fig. 2, Supplementary Table 3, 5, Supplementary Fig. 8).

### Decalcification and soft tissue imaging

All specimens were cleaned with ethanol before decalcification. For decalcification, 3 g of each sample were incubated in 2 mL Eppendorf tubes with 1.5 mL of a 0.1 M aqueous solution of hydrochloric acid (Merck, Germany) until complete structural disintegration was reached.

Only one-time use products, pipettes, glasses, and other equipment were used to avoid any systematic contamination. We used 150 mL of 2 M (bones and eggshells), and 38% (teeth) hydrochloric acid solutions purchased from Sigma Aldrich, poured in sterile, ethanol-cleaned 250 mL Kimax Pyrex sealable glass bottles. All samples were strictly separated during the procedure, and fossil and modern samples were never handled at the same time. Cleaned specimen samples were incubated in capped (to avoid any external contamination), but not fully sealed (to allow carbon dioxide release during decalcification) glass bottles. Incubation durations varied between specimens. Some fossil samples achieved complete structural disintegration in less than 10 min; other fossil and most modern samples required overnight incubation.

Following decalcification, released soft tissues were examined with a microscope (Fig. 1). Samples from oxidative environments^37^ yielded dark brown precipitates at the bottom of the glass bottle with a clear supernatant solution. All precipitates were washed with deionized water, and transferred into fresh 2 mL Eppendorf tubes filled with deionized water. Aliquots of all recovered precipitates were transferred onto ethanol-cleaned microscopic glass slides using one-time sterile plastic pipettes, and imaged in a CFscan mode using a petrographic 66 Leica DM 2500 P microscope equipped with a ProgRes 67 camera. Depending on size, soft tissue fragments were imaged at magnifications of ×10, ×20, ×50, or ×100 (immersion oil required). All microphotographs are white-balanced.

Decalcification of samples from reducing settings produced only dark supernatant solutions, but no precipitates. 1.5 mL aliquots of the decalcification solutions of these samples from reducing environments were centrifuged in 2 mL Eppendorf tubes at 8000× *g* for 15 min, to concentrate any microparticles floating in the solutions. In all cases, even centrifuging did not yield precipitates, indicating that hard tissues from reducing environments do not preserve soft tissue. All extracted fossil soft tissue samples were immediately subjected to Raman Microspectroscopy (*n* = 15). Modern hard tissue decalcification residues were treated in the same way as the fossil samples. Small aliquots of modern sample decalcification residues were subjected to experimental maturation at different temperatures and incubation times, followed by Raman Microspectroscopy.

### Experimental maturation

Experiments were designed to simulate protein alteration in deep time, and generation of AGE/ALE crosslinks^39^. Oxygen availability appears to be crucial for this process^39^. The experimental conditions promoted oxidation in the absence of external agents such as transition metal cations, phosphate anions, or large surface areas of sediment grains, and the experiments were carried out at low temperatures, which were likely experienced by fossil samples. We used 10 decalcified eggshell samples (*Rhea americana*), 10 decalcified bone samples (*Gallus domesticus*), and 9 decalcified enamel scales (*Lepisosteus osseus*) (due to sample limitations, *Lepisosteus osseus* scales were not subjected to the final stage of maturation). Soft tissues were extracted to expose them directly to oxidative reactions. Samples were each placed on separate, sterile microscopic glass slides. These glass slides were placed on heat plates at different temperatures: 25 °C (10 min), 45 °C (10 min), 60 °C (10, 20, 30 min), 90 °C (10, 20, 30 min), 120 °C (10, 20, 30, and 60 min). All soft tissue samples were continually moistened with deionized water. This experimental maturation leads to tissue browning and increase of the AGE/Amide I ratio (Figs. 2, 4, 5) consistent with the results of Hidalgo et al.^39^.

### Raman microspectroscopy and Chemospace analysis

Raman microspectroscopy was performed immediately after decalcification, in aqueous solution, using a Horiba LabRam HR800 in the Department of Geology and Geophysics at Yale University with 532 nm excitation (20 mW at the sample surface). The spectra were obtained in LabSpec 5 software (spectra acquisition, mapping acquisition, standard spike removal). The scattered Raman light was detected by an electron multiplying charge-coupled device (EM-CCD) after being dispersed with a 600 grooves/mm grating and passed through a 100 μm slit (hole size 300 μm). The spectrometer was calibrated using the first order Si band at 520.7 cm^−1^. Four spectra were accumulated in the 500–1800 cm^−1^ region for 20 s exposure time each (we additionally provide two full range—200–3000 cm^−1^—spectra of fossil soft tissues in Supplementary Fig. 6). All spectra were analyzed in SpectraGryph 1.2 spectroscopic software (adaptive baseline, 50%, no offset, minimally smoothed through rectangular averaging over an interval of 4 points) and an automated peak search was performed (threshold 5.00%, prominence 3). Raman maps were obtained (LabSpec 5 Software) from decalcified fossil soft tissues (*Allosaurus fragilis* bone, HCl-extracted extracellular matrix selected for its large size, *n* = 1) in aqueous solution (pH 3) at 1550–1610 cm^−1^, 980 cm^−1^, and 1690 cm^−1^ Raman shift, with a D 0.3 filter, at 3 s exposure with three accumulations. Raman bands characterizing the molecular composition of the decalcified hard tissues were selected for their intensity and exact Raman shift for each sample spectrum (including all fresh (*n* = 4), matured (*n* = 4), fossil (*n* = 7), and control samples (*n* = 13)), compiled in a data matrix (variance-covariance matrix), and subjected to a Principal Components Analysis in PAST 3 to generate a Chemospace (Fig. 4a, b). Average spectra from the fresh (*n* = 4) and fossil (*n* = 7) soft tissues were used to create a dissimilarity plot (Fig. 4c) through subtraction of the average spectrum of modern samples from the average spectrum of fossil samples (SpectraGryph 1.2). This dissimilarity plot illustrates trends in compositional changes resulting from fossilization.

## Electronic supplementary material

Supplementary Information

## Data Availability

The authors declare that the main data supporting the findings of this study are available within the main Article and Supplementary Figures 1–9. Sample materials are available from the corresponding author upon request.
