# Supplementary material for: Fossilization transforms vertebrate hard tissue proteins into N-heterocyclic polymers
Source: Nat Commun. 2018 Nov 9;9:4741. doi: 10.1038/s41467-018-07013-3 (PMC6226439; doi:10.1038/s41467-018-07013-3)
Supplement: Supplementary file 1 — Supplementary Information [file 41467_2018_7013_MOESM1_ESM.pdf]

Supplementary Information

Fossilization transforms vertebrate hard tissue proteins into N-heterocyclic polymers

Wiemann *et al.*

This Supplementary Information includes:

Supplementary Notes 1-4

1. Supplementary Note 1: Decalcification process
2. Supplementary Note 2: Raman band assignments
    a. Band assignments
    b. Staining minerals
    c. Glues, epoxy resins, and consolidant
    d. Humic acid
    e. Iron oxide/hydroxide as soft tissue composites
    f. Modern fauna-derived protein contamination
    g. Systemic contamination
    h. Sediment contamination
3. Supplementary Note 3: Samples from reducing environments
4. Supplementary Note 4: Chemospace PCA tests chemical similarity

Supplementary Figures 1-9
Supplementary Tables 1-7

Supplementary References

Supplementary Notes 1-4

1. Supplementary Note 1: Decalcification process

To confirm that the decalcification routine does not affect the chemistry of the soft tissues we investigated how the incubation of hard tissue with hydrochloric acid solution affects molecular preservation or AGE/ALE generation using *Allosaurus* bones to obtain high-resolution Raman spectra of *in situ* undecalcified and extracted decalcified samples. If incubation in hydrochloric acid solution induced crosslinks in the samples, the AGE/ALE band would be present in the decalcified sample but absent in the undecalcified sample. However, the AGE/ALE band heights were identical in the normalized spectra of undecalcified and decalcified samples (Supplementary Fig. 4). Peak labels shown in Supplementary Fig. 4 are obtained through the automatic peak label function (threshold 5.00 %, prominence 3) in SpectraGryph 1.2 spectroscopic software. The AGE/Amide I ratios were also identical, suggesting that hydrochloric acid incubation has no effect on the extracted proteins/peptides and their crosslinks, perhaps predicted by the fact that protein crosslinking is favored under alkaline, not acid, conditions[1,2]. The only Raman band induced by hydrochloric acid incubation is a C-Cl band at Raman shift 750-790 cm^{-1}. Generally, decalcification results in greater resolution of the Raman bands in a soft tissue sample.

An additional implication of the comparison between undecalcified and decalcified *Allosaurus* bone is the prominence of organic AGE/ALE and amide I bands in the undecalcified spectrum. Such an analysis offers a nondestructive assessment of proteinaceous/peptide soft tissue preservation in a fossil hard tissue.

Raman Spectroscopy of undecalcified samples from reducing settings yielded no evidence for soft tissue material (see Supplementary Fig. 9).

2. Supplementary Note 2: Raman band assignments

a. Band assignments

Our Raman band assignments (Supplementary Tab. 6), which are based on published data[2,3,4-13], are conservative to avoid overinterpretation. The prominent AGE/ALE band, which represents a key observation in our study, corresponds with the amide II band which indicates tryptophane-like compounds. The broad mixture of different diagenetic AGEs/ALEs increases the band width due to addition of different imidazole-like ring vibrations. We interpret this band as the result of a minor contribution of peptide tryptophane residues in the fresh, matured, and preserved fossil soft tissues. Our artificial maturation experiments clearly indicate a major contribution of similar, secondary pyrrole, imidazol(on)e-like structures generated by the reaction of amino acid residues (Fig. 5). These structures generated during artificial maturation appear identical in composition to protein degradation products observed in hard tissue fossils. Representative spectra covering the full wavenumber range for fossil *Allosaurus* bone and fossil *Heyuannia huangi* eggshell are shown for completeness (Supplementary Fig. 6).

b. Staining minerals

We compared the spectra obtained from our undecalcified and decalcified extant, matured, and fossil tissues (from oxidative and reducing settings) with published mineral

spectra in the RRuff database[14]. We focused on pyrite, hematite and related oxo-hydroxo-iron compounds (e.g., goethite and fluorapatite, which occurs in different colors due to trace metal incorporation and crystal lattice defects), to investigate hard tissue discoloration due to staining minerals (Supplementary Fig. 7). Peak labels shown in Supplementary Fig. 7 were obtained through the automatic peak label function (threshold 5.00 %, prominence 3) in SpectraGryph 1.2 spectroscopic software. Pyrite was absent in all modern, matured, and fossil soft-tissue yielding samples (thus, samples from oxidative environments). Fluorapatite, the diagenetic product of hydroxyapatite, was present only in the undecalcified extant, matured, and fossil samples from both reducing and oxidative settings, and there were no significant band shifts indicating trace metal substitutions in the crystal lattice. Hematite and related iron compounds were found as traces in the undecalcified fossil samples from oxidative settings but were absent in the decalcified samples from oxidative settings yielding soft tissues. The extracted fossil soft tissues exhibited a significant dark brown stain but yielded only organic Raman signatures and no evidence of staining mineral compounds. The correlation between discoloration intensity and AGE/ALE concentration (Fig. 1) indicates that the observed discoloration in matured and fossil soft tissues is organic in origin.

c. **Glues, epoxy resins, and consolidant**

In order to test for contaminants which might produce soft tissue morphologies, or account for the observed amide bands, we analyzed Araldite epoxy resin, polyacrylamide glue, and acrylamide-based consolidant, all compounds used in the conservation of fossil bone. Polymeric acrylamide-based glues or consolidants are the only compounds used in

vertebrate fossil conservation that might result in Raman peaks that could be confused with proteins (since they are polyacrylamides). The spectra obtained from glue, epoxy, and consolidant (Supplementary Fig. 7), however, are very different. We also subjected all glue and epoxy samples to the same hydrochloride acid incubation as the tissue samples, and likewise obtained different signatures. We used these spectra to determine whether consolidant, glues, or epoxy were applied to our samples, complementing visual observations.

Raman spectra of these contaminants included many more narrow bands than the soft-tissue samples (Supplementary Fig. 7). We identified three marker bands at 630, 830, and 1111 cm^{-1} which we used to identify glue, epoxy, or consolidant contaminated samples in our data set. This revealed glue in two samples, the *Maiasaura* bone and the ichthyosaur bone, both from reducing environments, which did not produce any soft tissues after decalcification, only small, transparent glue sheets of random 3-dimensional shape. The spectra from these samples included peaks for all three types of glue. We retained these "contaminated" specimens in our data set to ensure the reliability of our screening for consolidants (see Supplementary Fig. 7). None of our extracted soft tissues showed evidence of glue, epoxy, or consolidant. Thus, we can rule out the possibility that the extracted soft tissues represent polyacrylamide-based glue "casts" of voids such as osteocyte lacunae or Haversian canals.

d. **Humic acid**

The archeological literature provides many examples of bones buried for less than 100,000 years that exhibit a black stain due to humic acid impregnation[15,16]. Humic acids,

as well as humic colloids, are abundant in coals, peats, and especially soils. They are polymeric carboxylic, carbonylic, and phenolic compounds which exhibit a brownish stain and have been extensively analysed using Raman spectroscopy[17]. Humic acids, in contrast to AGEs/ALEs, do not generate amide bands and show no significant C-N, imidazole or pyrrole vibrations. They are characterized mainly by two distinct bands, one at a Raman shift of 1308 cm^{-1} with a shoulder at 1251 cm^{-1}, and a second at 1611 cm^{-1} [17]. Such a signature is not present in any of our extracted fossil soft tissues. Only two samples produce spectra approaching a strongly peroxidized, humic acid-related composition. Soft tissue from the eggshell of *Psammornis rothschildi* exhibits a strongly reduced Raman spectrum including a significant AGE/ALE band based on C-N derivatives, which are not a significant presence in humic acids/humic colloids. The black shale matrix of the Jurassic *Ichthyosaurus* bone did not produce any soft tissue morphologies, but neither does it show the Raman band shifts characteristic of humic acids. Thus, our fossil soft tissues do not contain humic acid colloids.

e. **Iron oxide/hydroxide as soft tissue composites**

It has been suggested that iron oxides and hydroxides "coat" voids produced by soft tissue decay in fossil hard tissues[18]. This was suggested based on comparison of SEM images of a putative extracted, fossil vascular canal surface, and morphologically similar structures replicated by pyrite framboids[18]. Iron hydroxyoxides occur as colloids in soils and may percolate fossil bones. However, biofilm-associated iron oxides filling voids in hard tissue would not explain amide signals that are not associated with a biofilm (see Fig. 2). In any case, iron oxides/hydroxides are not revealed by our Raman analysis

(characteristic bands at 370 to 400 cm^{-1} [29] are absent) and cannot explain the organic peptide signatures in the fossils.

f. Modern fauna-derived protein contamination

The soft tissues extracted from fossils exhibit very different properties to those of the soft tissues from modern hard tissues. In contrast to the smooth, homogenous, and colorless transparent modern soft tissues, fossil soft tissues are brittle and show cracked surfaces and intense brown staining (not caused by decalcification treatment, see Supplementary Fig. 3). Moreover, the fossil soft tissues exhibit a chemical composition similar to that of artificially matured modern soft tissues, but significantly different from modern soft tissues or bacterial biofilms[19,20]. Studies of bacterial biofilms[21,22] revealed the presence of bacterial chromophores[19,20], which are absent in all our extracted fossil soft tissues (there is no evidence of carotenoids, which give rise to clear bands at 1152 and 1502 cm^{-1}). Thus we can discount the possibility that our extracted fossil soft tissues are a product of modern contamination (contra[18]).

g. Systemic contamination

We analyzed extracted modern, matured, and fossil soft tissues, and undecalcified *in situ* samples (Supplementary Fig. 9), as well as glue, consolidant, epoxy resin, and sediment samples. The restriction of proteinaceous/peptide AGEs/ALEs to matured modern soft tissues and fossil soft tissues from oxidative environments is clear evidence that our samples were not contaminated by a laboratory source of crosslinked proteins.

h. **Sediment contamination**

Dark sediment from reducing environments generally contains significant organics which are a potential source of contamination of contained fossil hard tissues. To test this possibility in our samples, we subjected sediment samples to *in situ* Raman Spectroscopy and compared the spectra with those of extracted soft tissues (Supplementary Fig. 8). Peak labels shown in the Supplementary Fig. 8 are obtained through the automatic peak label function (threshold 5.00 %, prominence 3) in SpectraGryph 1.2 spectroscopic software. Decalcification of hard tissues does not affect the chemistry of the soft tissues released (Supplementary Fig. 4). Neither the red sandstone sediment associated with the *Heyuannia huangi* eggshell from China nor the grey calcareous sandstone associated with *Saltasaurus* eggshell from Argentina contain significant organic material. The beige-greyish sandstone matrix associated with the diplodocid bone contains visible organic plant matter. These plant remains give rise to signatures indicative of peroxidized lipids and/or proteins in the Raman spectra, which are similar, but not identical, to signatures obtained from the fossil osteocytes and blood vessels extracted from the bone (Fig. 4a). Both oxidative protein crosslinking and lipoxidation have been proposed to affect plant organic matter[22]. The peptide signal in the plant material associated with the diplodocid bone is much less pronounced than the amide bands obtained from the fossil soft tissues extracted from the bone itself. This contrast can be explained by compositional differences and the effect of the envelope of hard tissue (bone) which provided a chemical and mechanical shield for the soft tissues within. The occurrence of abundant soft tissues in hard tissues from sediment containing abundant fossil plant remains cannot be explained by contamination from the plants. All the dark colored sediments

investigated probably contain minor amounts of degraded organics (derived from plant matter, algae, etc.) which contribute to the color, but there is no evidence of significant organic signatures in their Raman spectra. Dark mudstones and black shales contain higher concentrations of organics which appear to be highly degraded and produce almost humic acid-like chemical signatures with a preponderance of C-N rich functions rather than C-O functions[23-25]. However, Raman spectra of such sediment samples do not reveal peptide bands, and neither do the hard tissues in these sediments yield soft tissues or peptide signals. The absence of carboxyl and carbonyl functions in these sediment organics is consistent with the reducing environments[1] that dark mudstones and shales represent. The degraded nature of these matrix organics demonstrates that reducing environments do not promote preservation of endogenous proteinaceous matter.

3. Supplementary Note 3: Samples from reducing environments

Undecalcified fossil hard tissues from reducing environments[1], which did not produce soft tissues during decalcification, did not yield amide signals and do not contain proteins/peptides. Nevertheless, *in situ* analyses of some undecalcified fossil hard tissues (Supplementary Fig. 9) revealed lipids, which may be preserved via oxidative crosslinking[27] and produce characteristic Raman bands of unaltered lipid chains around 1440-1450 cm^{-1}, 1650-1690 cm^{-1}, and 1720-1750 cm^{-1}. Fossil lipids also produce an ALE band in the Raman shift area of the major AGEs/ALEs at 1550-1600 cm^{-1} (Supplementary Tab. 6). This was evident in the Cretaceous *Iguanodon* bone and the Triassic *Nothosaurus* tooth. Neither of these samples produced any amide signals or soft tissues during decalcification, but showed all three lipid bands plus an ALE band. The

Nothosaurus tooth was preserved in a mildly reducing[1] grey sandstone from the otherwise largely oxidative Lettenkeuper sediments in Germany (see Supplementary Tab. 5), but the *Iguanodon* bone was preserved in a strongly reducing, pyrite-rich dark mudstone (see Supplementary Tab. 5). The mildly reducing conditions explain the strong lipid signals derived from the *Nothosaurus* tooth, and the lack of the less resilient peptides. Lipids/ALEs were also recovered from the *Maiasaura* and *Saltasaurus* specimens, which occur in sediments largely free of organics. It appears that crosslinked lipids can preserve in reducing environments, in contrast to proteinaceous soft tissues. A comparison of the decay resistance of fossil ALE/lipids versus AGE/ALE/peptides is a subject for future investigation. Even though ALEs/lipids are preserved in some specimens from dark sediments, indicating reducing settings, peptide signals were not evident and no soft tissue was released by decalcification. Peak labels shown in Supplementary Fig. 9 are obtained through the automatic peak label function (threshold 5.00 %, prominence 3) in SpectraGryph 1.2 spectroscopic software.

4. Supplementary Note 4: Chemospace PCA tests chemical similarity

We quantified the similarity among the spectra obtained for the fresh, matured and fossil material using Principal Component Analyses in PAST 3 (Fig. 4). We also included control samples: fossils preserved in reducing environments, and chemical compounds such as glues and epoxy, which may have been introduced during preparation and conservation (Supplementary Figs. 7, 8). As previously proposed[26,27], characteristic peaks can be extracted from the spectra to perform PCA. We selected 15 Raman shift ranges (Supplementary Tab. 7) representing the molecular composition of tissues using the

software SpectraGryph 1.2. Before extraction of the peaks, spectra were subjected to baseline correction and normalization. We extracted normalized intensity values at these band positions and compiled them in a data matrix to be used for ChemoSpace analytics. The data show a normal distribution.

PC1 represents 56.93% of the overall variance, when control samples are included, and 50.19% when control samples are excluded. PC1 represents the crosslink bands 880 cm^{-1}, 1400-1430 cm^{-1}, 1434-1440 cm^{-1}, and 1515-1520 cm^{-1}. PC2 explains 10.49% of the variance when control samples are included and 19.45% when they are excluded. PC2 represents the amide bands 1230-1250 cm^{-1}, 1650-1690 cm^{-1}, the crosslink band 1550-1600 cm^{-1}, and the peptide band 1360-1370 cm^{-1} (compare Supplementary Tab. 6). Overall, control samples do not overlap with modern, matured or fossil material (Fig. 3a). The modern samples occupy a separate area of the chemospace with no overlap with fossils, whereas the artificially matured samples fall in an intermediate region (Fig. 3b). Eggshell material is markedly different from the other types of hard tissues and shows a different trajectory of maturation and AGE formation. The occupation of the chemospace shows no correlation with geological age or phylogenetic position, demonstrating that burial history and environment are the main factors determining soft tissue preservation. The overlap of the artificially matured samples with the fossil material in the chemospace shows that the experiments mimic the chemical mechanism for molecular preservation. This result shows that oxidative environments, and minor temperature overprinting are key variables in the degradation of proteinaceous material and formation of AGEs in hard tissues, and for the preservation of soft tissues and their constituent molecules in the fossil record.

Supplementary Figures 1-9

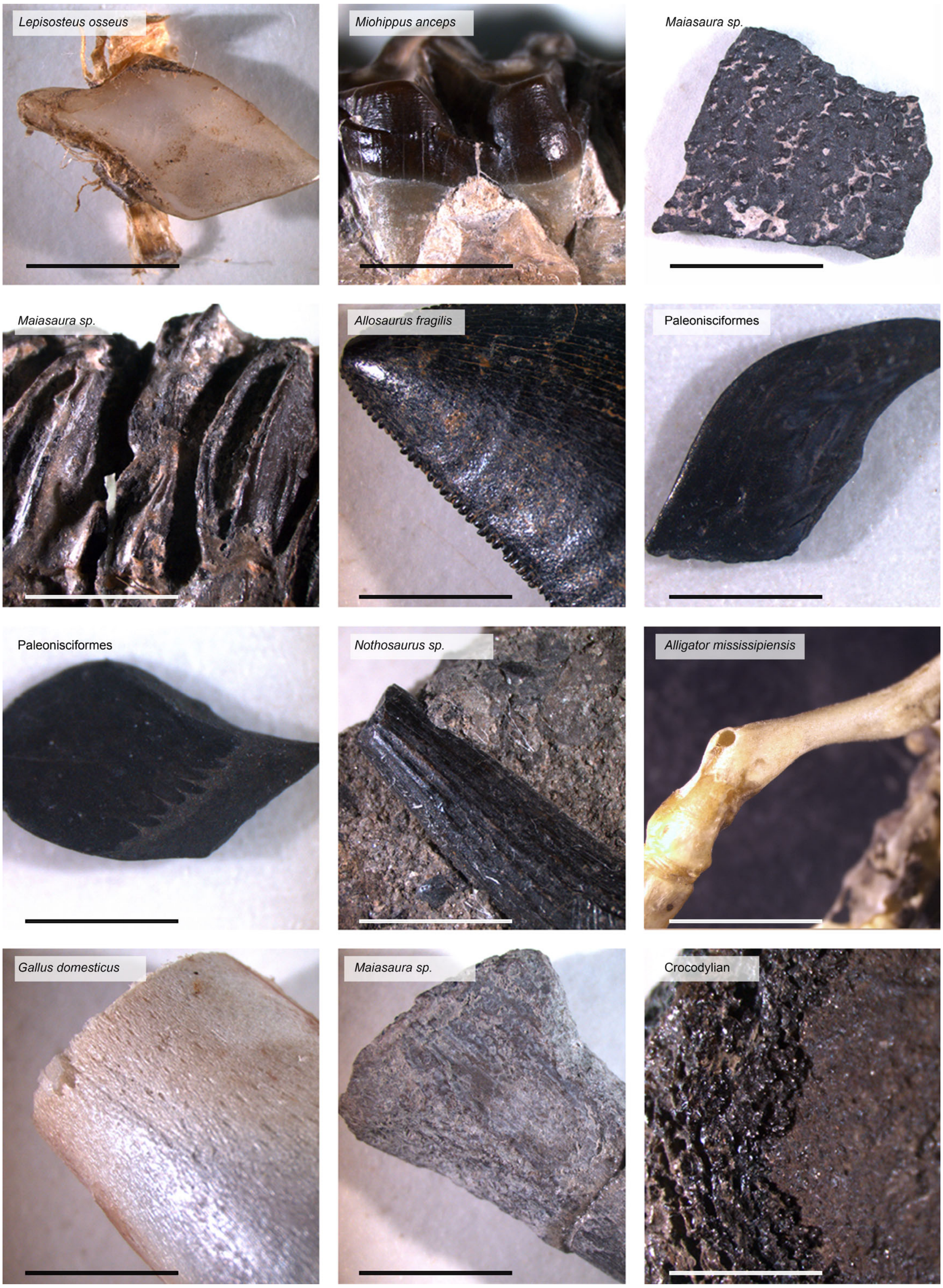

Supplementary Figure 1: Sampled specimens. See Supplementary Tables 1-4 for specimen information. The scale bar represents 5 mm.

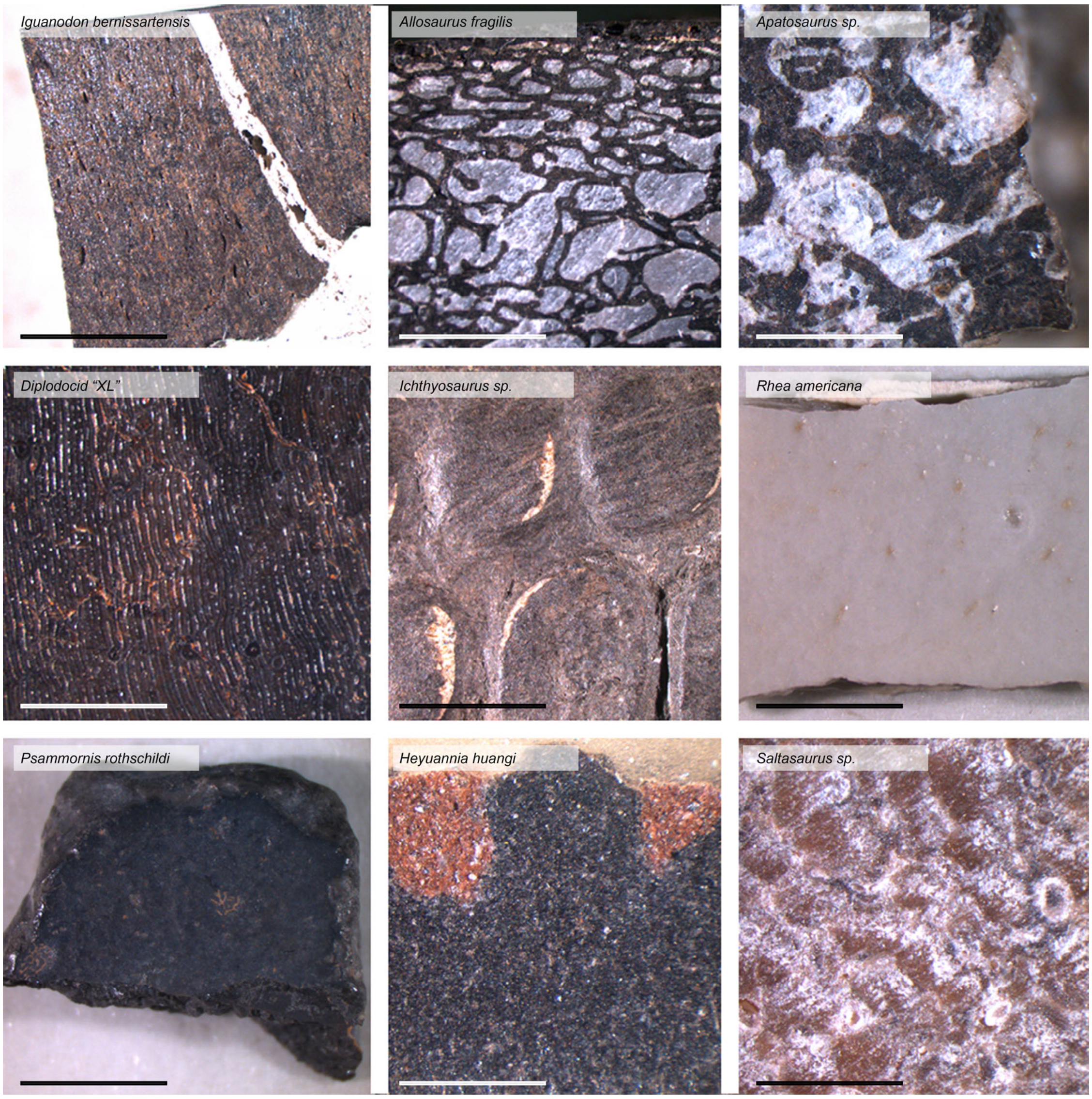

Supplementary Figure 2: Sampled specimens. See Supplementary Tables 1-4 for specimen information. The scale bar represents 1 mm.

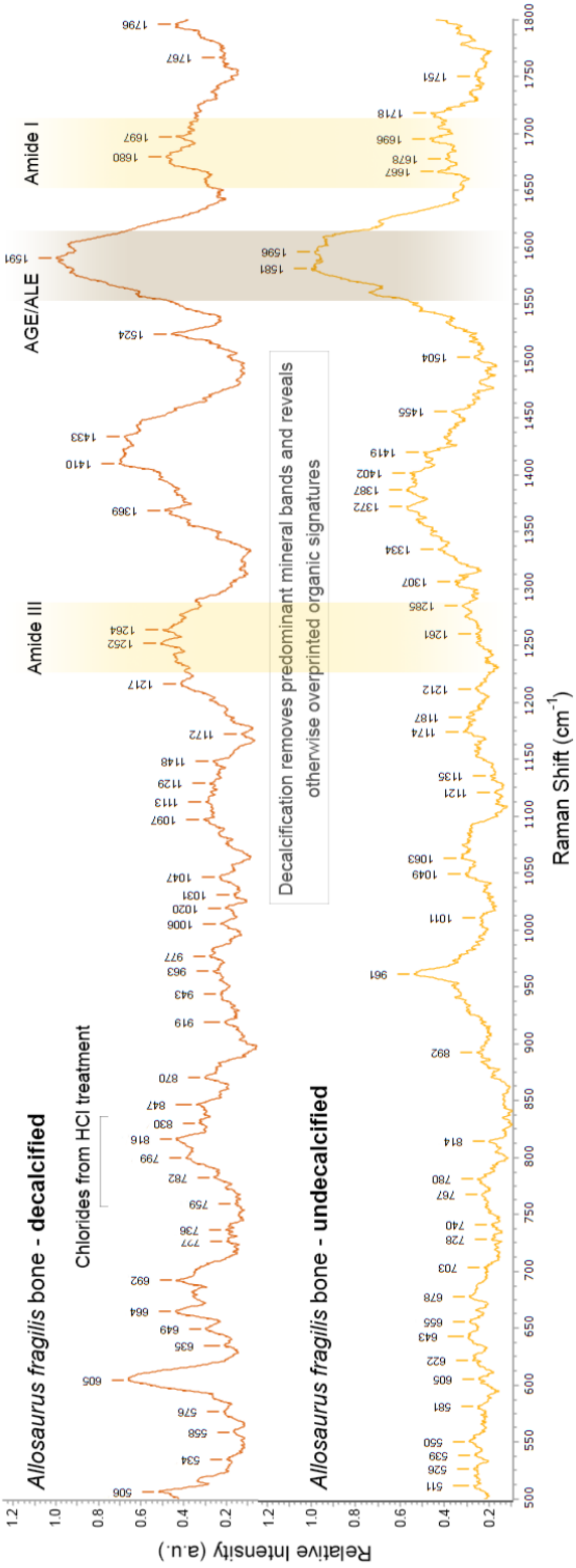

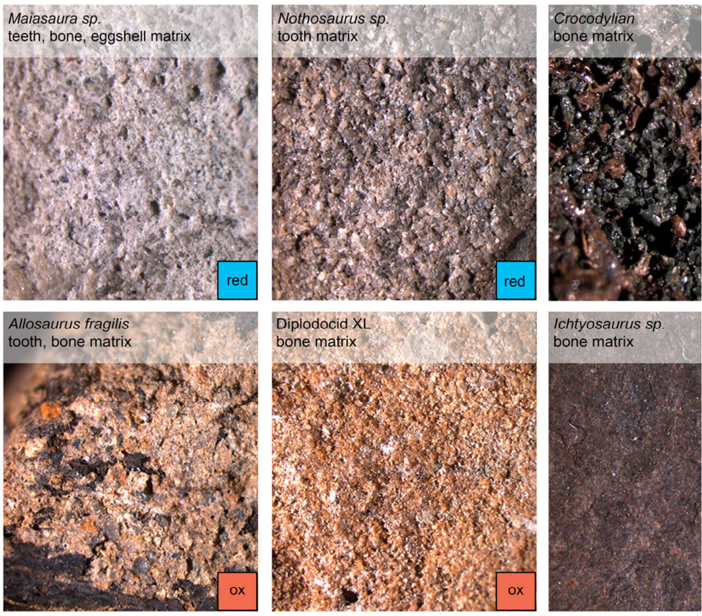

Supplementary Figure 3: Associated sediment samples (as available). See Supplementary Tables 1-4 for specimen information. red = reducing environment; ox = oxidizing environment.

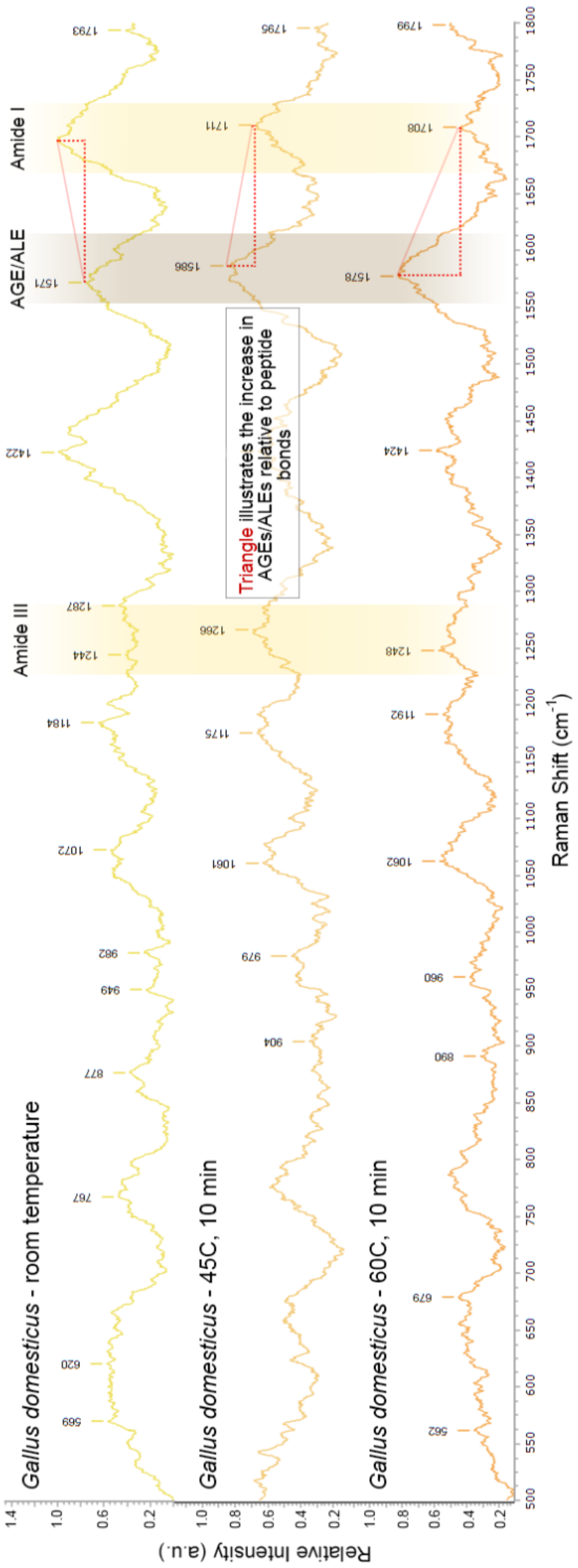

Supplementary Figure 6: Full range spectra for two representatives of fossil

material containing preserved soft tissues.

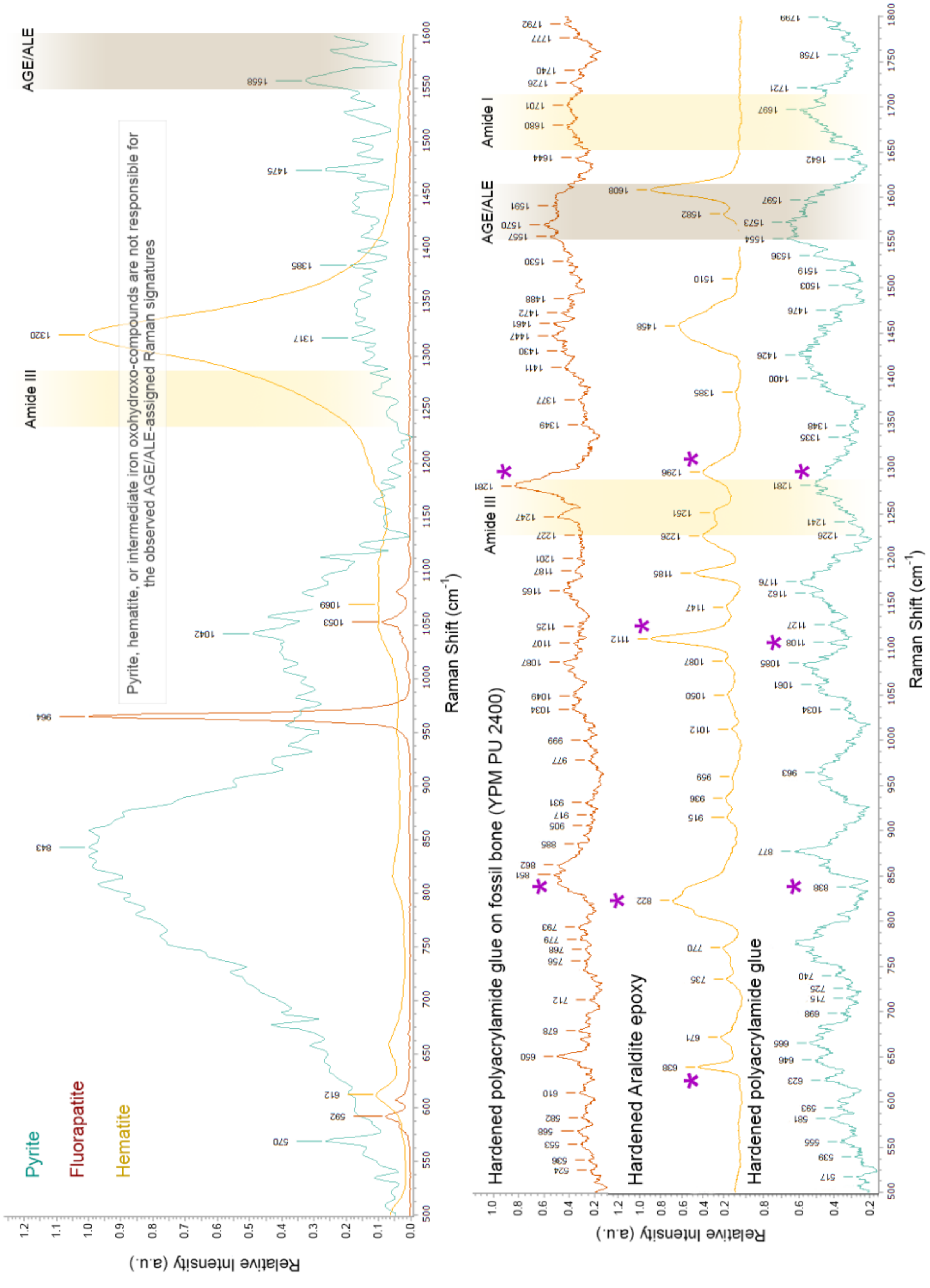

Supplementary Figure 7: Stacked Raman spectra of the potentially staining minerals and glues. **a,** Pyrite, Fluorapatite, Hematite. Yellow bands indicate amide III and I bands; brown bands label the Raman shift of AGE/ALEs. **b,** Stacked Raman spectra of hardened epoxy resin, polyacrylamide on bone, and hardened polyacrylamide glue. The purple asterices label glue/epoxy resin/consolidant markers, the yellow bands indicate amide III and I bands, and the brown band labels the Raman shift of AGE/ALEs.

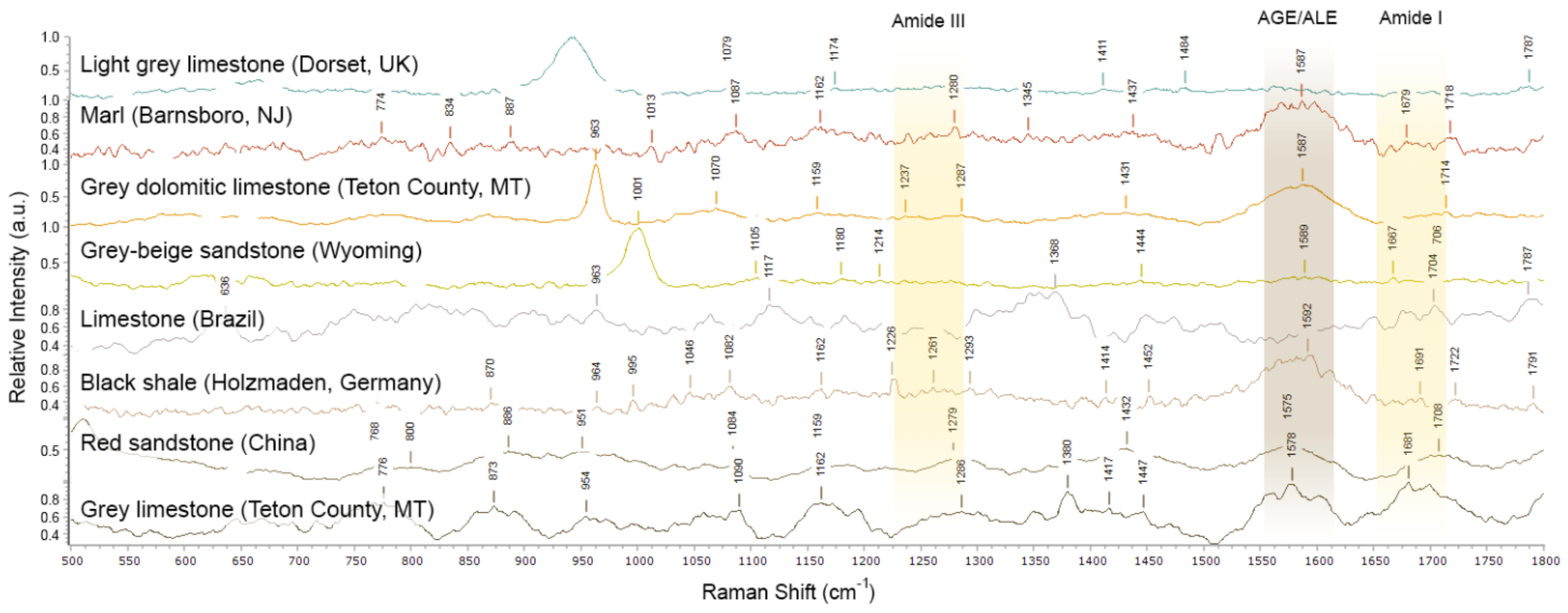

Supplementary Figure 8: Stacked Raman spectra of oxidative and reducing sediment samples (compare Supplementary

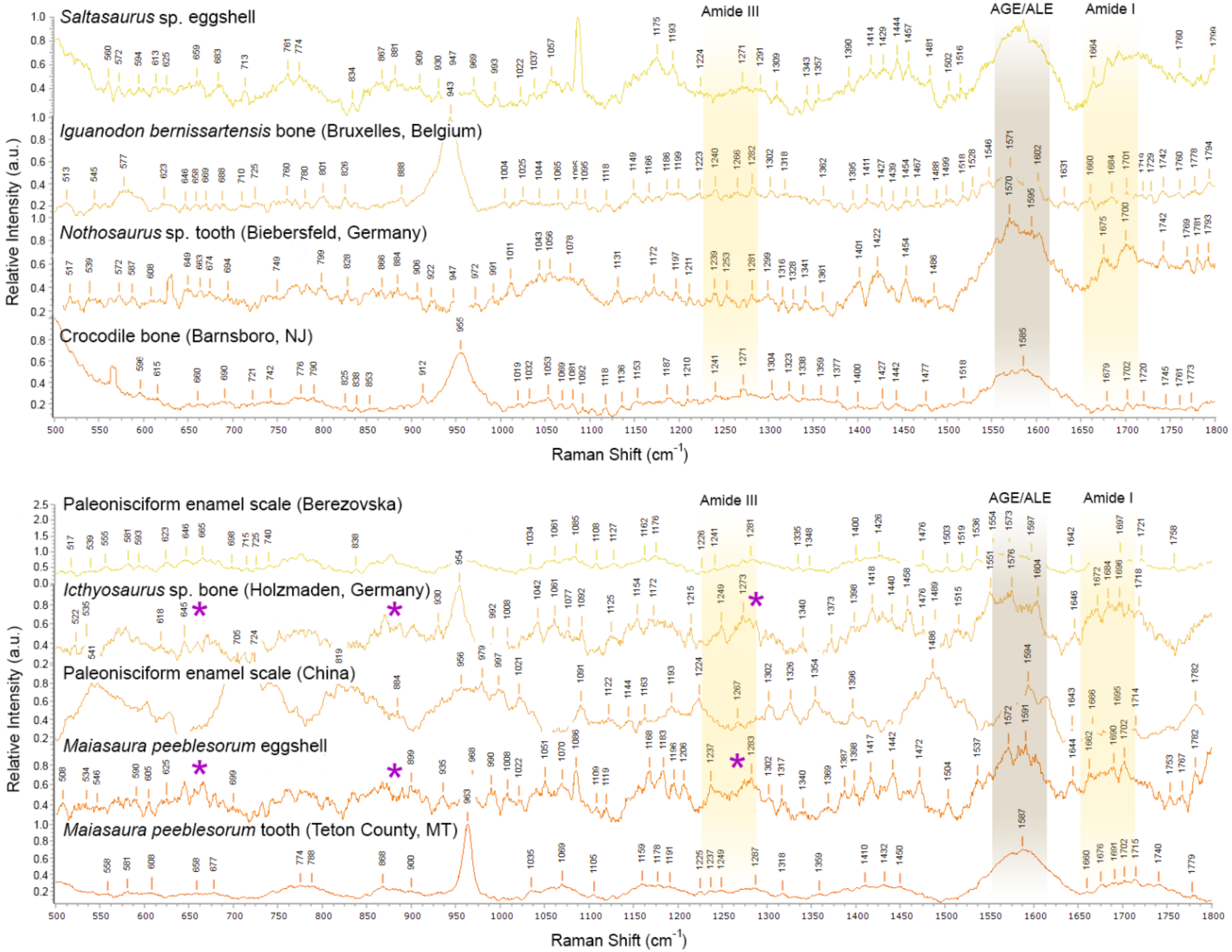
Saltasaurus sp. eggshell
Iguanodon bernissartensis bone (Bruxelles, Belgium)
Nothosaurus sp. tooth (Biebersfeld, Germany)
Crocodile bone (Barnsboro, NJ)
Amide III
AGE/ALE
Amide I
Relative Intensity (a.u.)
Raman Shift (cm-1)
Paleonisciform enamel scale (Berezovska)
Icthyosaurus sp. bone (Holzmaden, Germany)
Paleonisciform enamel scale (China)
Maiasaura peeblesorum eggshell
Maiasaura peeblesorum tooth (Teton County, MT)
Amide III
AGE/ALE
Amide I
Relative Intensity (a.u.)
Raman Shift (cm-1)

Supplementary Figure 9: Stacked Raman spectra of undecalcified hard tissues from reducing environments (compare Supplementary Table 1-4). The *Iguanodon* bones, *Nothosaurus* bones, and Maiasaura teeth contain lipid ALEs, but no amides indicating proteins/peptides. The *Maiasaura* bones, eggshells, and the *Ichthyosaurus* bones were treated with consolidants. Lipid and ALE band positions are labelled with purple asterisks. In the *Saltasaurus* eggshells, whewellite, a calcium oxalate, might contribute to the ALE Raman band with its abundant COO^- anions.

Supplementary Tables 1-7

Supplementary Table 1: Specimen data, catalogue numbers, and ages. Teeth and enamel scales, bones, and eggshells. Artificially matured samples are labeled in the taxon column with an asterisk*. Collection acronyms: BYU, Museum of Ancient Life, Brigham University, Provo, US; NMNS, National Museum of Natural Science, Taichung, Taiwan; RBINS, Royal Belgian Institute of Natural Science, Brussels, Belgium; SMA, Sauriermuseum Aathal, Aahthal-Seegraeben, Switzerland; STIPB, Steinmann Institute, University of Bonn, Bonn, Germany; YPM, Yale Peabody Museum, Yale University, New Haven, US; ZFMK, Zoological Research Museum Koenig, Bonn, Germany.

Specimen	Catalogue number	Age
Lepisosteus osseus	YPM ICH 804	Recent
*Lepisosteus osseus**	YPM ICH 804	Matured
Miohippus anceps	YPM 33780	Tertiary
Maiasaura sp.	YPM PU 22523	Cretaceous

Allosaurus fragilis	YPM 48	Late Jurassic
Paleonisciform fish	STIPB F25556	Late Jurassic
Paleonisciform fish	STIPB F25557	Late Jurassic
Nothosaurus sp.	YPM 2222	Late Triassic
Alligator mississippiensis	YPM 759	Recent
Gallus domesticus	Uncatalogued	Recent
*Gallus domesticus**	Uncatalogued	Matured
Maiasaura sp.	YPM PU 22400	Cretaceous
Crocodilian	YPM 656	Cretaceous
Iguanodon bernissartensis	RBINS I7	Early Cretaceous
Allosaurus fragilis	YPM 48	Late Jurassic
Apatosaurus sp.	BYU681-16782	Late Jurassic
Diplodocid	SMA G44/96-1	Late Jurassic
Ichthyosaurus sp.	YPM VP 059362	Early Jurassic
Rhea americana	Uncatalogued	Recent
*Rhea americana**	Uncatalogued	Matured
Psammornis rothschildi	ZFMK 23a: III/c/23	Holocene
Heyuannia huangi	NMNS CYN-2004-DINO-05	Late Cretaceous
Saltasaurus sp.	STIPB E360	Late Cretaceous
Maiasaura sp.	YPM PU 22523	Cretaceous

Supplementary Table 2: Specimen data, localities, and sedimentology. Artificially matured samples are labelled in the taxon column with an asterix*.

Specimen	Locality	Sedimentology
Lepisosteus osseus	St. John's River, FL, USA	Sand
*Lepisosteus osseus**	St. John's River, FL, USA	Sand
Miohippus anceps	John Day, OR, USA	Light sandstone

Maiasaura sp.	Two Med. Fm., MT, USA	Grey mudstone
Allosaurus fragilis	Carbon County, WY, USA	Beige sandstone
Paleonisciform fish	Qigu Fm., Xinjiang, China	Red sandy siltstone
Paleonisciform fish	Itat Fm., Siberia, Russia	Dark mudstone, coals
Nothosaurus sp.	Biebersfeld, Germany	Grey sandstone
Alligator mississippiensis	Jacksonville, FL, USA	Soil
Gallus domesticus	New Haven, CT, USA	None
*Gallus domesticus**	New Haven, CT, USA	None
Maiasaura sp.	Two Med. Fm., MT, USA	Grey mudstone
Crocodilian	Barnsboro, NJ, USA	Green sandstone, dark marl
Iguanodon bernissartensis	Bernissart, Belgium	Pyrite-rich dark mudstone
Allosaurus fragilis	Carbon County, WY, USA	Beige sandstone
Apatosaurus sp.	Morrison Fm., CO, USA	Beige sandstone
Diplodocid	Howe-St. Quarry, WY, USA	Beige sandstone
Ichthyosaurus sp.	Reutlingen, Germany	Black shale
Rhea americana	Bozeman, MT, USA	Soil
*Rhea americana**	Bozeman, MT, USA	Soil
Psammornis rothschildi	Djokran, Algeria	Red dune sands
Heyuannia huangi	Yuanpu Fm., Jiangxi, China	Red sandstone
Saltasaurus sp.	La Rioja, Argentina	Grey calcite-cem. sandstone
Maiasaura sp.	Two Med. Fm., MT, USA	Grey mudstone

Supplementary Table 3: Specimen data, minerals, and AGE/amide I ratio of extracted soft tissues. The minerals detected are HA = hydroxyapatite, C = calcite, FA = fluorapatite, Cl = clay minerals, Py = pyrite, B = baryte, and G = gypsum (may be a

contaminant from plaster jacket). The presence of amides is diagnostic for peptides/proteins. AGE/Amide I = Advanced glycoxidation endproduct (AGE, Raman band 1550-1600 cm^{-1}) to amide I (peptide marker, Raman band 1650 – 1690 cm^{-1}) ratio. Increasing AGE/Amide I ratios correspond with the increase in brown staining of the tissue organics (see Supplementary Tab. 7). When no soft tissues were released by decalcification no AGE/amide I ratio was obtained. Artificially matured samples are labelled in the taxon column with an asterisk*.

**Specimen**	**Sample**	**Minerals**	**AGE/Amide I ratio**
Lepisosteus osseus	Enamel scale	HA	0.8986
*Lepisosteus osseus**	Enamel scale	HA	0.9426
Miohippus anceps	Tooth	C, FA	1.0968
Maiasaura sp.	Tooth	C, FA, B, G, Cl	Not available
Allosaurus fragilis	Tooth	C, FA	1.4144
Paleonisciform fish	Enamel scale	C, FA	1.8286
Paleonisciform fish	Enamel scale	C, FA, Cl, Py	Not available
Nothosaurus sp.	Tooth	FA, Cl	Not available
Alligator mississippiensis	Femur	HA	0.7734
Gallus domesticus	Femur	HA	1.2372
*Gallus domesticus**	Femur	HA	2.0803
Maiasaura sp.	Femur	C, FA, Cl	Not available
Crocodilian	Vertebra	C, FA, Cl	Not available
Iguanodon bernissartensis	Femur	C, FA, Py, Cl	Not available
Allosaurus fragilis	Vertebra	C, FA	1.4144
Apatosaurus sp.	Femur	C, FA	Not available
Diplodocid	Ulna	C, FA	1.9565
Ichthyosaurus sp.	Phalanx	C, FA, Py	Not available

Rhea americana	Eggshell	C, HA	1.2727
*Rhea americana**	Eggshell	C, HA	1.2963
Psammornis rothschildi	Eggshell	C, HA	2.4444
Heyuannia huangi	Eggshell	C, HA	1.7467
Saltasaurus sp.	Eggshell	C, B, G, Whw	Not available
Maiasaura sp.	Eggshell	C, G, G, Cl	Not available

Supplementary Table 4: Specimen data, and extracted RGB colour values of soft tissues. When no soft tissues were released by decalcification no soft tissue RGB color value was obtained. Artificially matured samples are labelled in the taxon column with an asterisk*.

Specimen	Soft Tissue Colour (Extracted RGB Values)		
Lepisosteus osseus	Transparent		
*Lepisosteus osseus**	R 229	G 212	B 197
Miohippus anceps	R 156	G 132	B 89
Maiasaura sp.	Not available		
Allosaurus fragilis	R 37	G 0	B 0
Paleonisciform fish	R 38	G 27	B 25
Paleonisciform fish	Not available		
Nothosaurus sp.	Not available		
Alligator mississippiensis	Transparent		
Gallus domesticus	Transparent		
*Gallus domesticus**	R 176	G 153	B 126
Maiasaura sp.	Not available		
Crocodilian	Not available		
Iguanodon bernissartensis	Not available		

Allosaurus fragilis	R 87	G 12	B 5
Apatosaurus sp.	R 54	G 51	B 40
Diplodocid	R 80	G 40	B 20
Ichthyosaurus sp.	Na		
Rhea americana	Transparent		
*Rhea americana**	R 170	G 144	B 106
Psammornis rothschildi	R 94	G 75	B 68
Heyuannia huangi	R 115	G 101	B 89
Saltasaurus sp.	Not available		
Maiasaura sp.	Not available		

Supplementary Table 5: Depositional environments of the investigated specimens.

Specimen	Publication	Sediments
Miohippus anceps	Woodburne & Robinson[28]	Light tuffaceous sandstone & siltstone
Maiasaura sp.	Oser & Jackson[29]	(Parauthochthonous) grey mudstone
Allosaurus fragilis	Dalman[30], Dodson et al.[31]	Beige, reddish sandstone
Paleonisciform fish (China)	Martin et al.[32]	Reddish siltstone
Paleonisciform fish (Russia)	Martin et al.[33]	Dark mudstone, above coal
Nothosaurus sp.	Hagdorn et al.[34]	Grey sandstone (Lettenkeuper)
Maiasaura sp.	Oser & Jackson[35]	(Parauthochthonous) grey mudstone
Crocodylian	Cope[36]	Green sandstone, brown marl
Iguanodon bernissartensis	Schnyder et al.[37]	Dark mudstone
Allosaurus fragilis	Dalman[30], Dodson et al.[31]	Beige, reddish sandstone
Apatosaurus sp.	Kirkland et al.[38], Balanoff et	Beige sandstone

	al.[39], Dodson et al.[31]	
Diplodocid XL	Siber & Moeckli[40], Dodson et al.[31]	Beige sandstone with fossil, organic plant matter (carbonized)
Ichthyosaurus sp.	Röhl et al.[41]	Black shale (Posidonia Shale)
Psammornis rothschildi	Sauer[42]	Red dunesand
Heyuannia huangi	Liang et al.[43]	Red sandstone
Saltasaurus sp.	Grellet-Tinner & Fiorelli[44]	Calcite-cemented sandstone, hydrothermal overprinting, rhizoliths
Maiasaura sp.	Oser & Jackson[35]	(Parauthochthonous) grey mudstone

Supplementary Table 6: Raman band assignments.

Raman Shift	Band Assignment	Details
1650-1690	Peptide (Amide I)	C=O stretch
1550-1600	AGE/ALE	Pyridine-like ring stretch
1550-1600	(Peptide)/AGE/ALE	C=C stretch in (metal) imidazole ring
1515-1520	AGE/ALE	N-H deformation
1430-1440	Peptide	CH_3 antisymmetric deformation
1434-1440	(Peptide)/AGE/ALE	(Metal) imidazole ring vibration
1400-1430	(Peptide)/AGE/ALE	C-N stretch
1360-1370	Peptide	CH_3 symmetric deformation
1340-1352	(Peptide)/AGE/ALE	(Metal) imidazole ring vibration
1230-1250	Peptide (Amide III)	C-C-N bending, C-N stretch, N-H bend
1170-1180	Peptide	C-O-C stretch
1080-1140	AGE/ALE	C-N stretch
1050-1070	AGE/ALE	C-O stretch

980	Peptide/AGE/ALE	CH_2=CH_2 out-of-plane deformation
960	Peptide/AGE/ALE	CH out-of-plane deformation
908	Peptide/AGE/ALE	CH_2 out-of-plane wagging
880	Peptide/AGE/ALE	CH_2 out-of-plane wagging
830-840	Peptide/AGE/ALE	NH_2 stretch
750-790	Acid Extraction	C-Cl stretch
650-680	Peptide/AGE/ALE	N-C=O bend
550	Peptide	Disulfide bridge
520	Peptide	Disulfide bridge
500	Peptide	Disulfide bridge

Raman Shift	Band Assignment	Details
1720-1750	Lipids	C=O stretch
1650-1660	Lipids	C=C stretch
1440-1460	Lipids	CH_3 antisymmetric deformation

Supplementary Table 7: Raman bands selected for the PCA (Fig. 4) and their assignments.

Raman Band	Band Assignment
510	Peptide
630	Peptide/AGE/ALE
803	Peptide/AGE/ALE
830	Peptide/AGE/ALE
880	Peptide/AGE/ALE
980	Peptide/AGE/ALE
1080	AGE/ALE
1230-1250	Peptide/Amide III
1340-1352	(Peptide)/AGE/ALE
1360-1370	Peptide

1400-1430	(Peptide)/AGE/ALE
1434-1440	(Peptide)/AGE/ALE
1450-1460	Peptide
1515-1520	AGE/ALE
1550-1600	(Peptide)/AGE/ALE
1650-1690	Peptide/Amide I
1720-1750	Lipids

Supplementary References

1. Myrow, P. M. A new graph for understanding colors of mudrocks and shales. *J. Geol. Edu.* **28**, 16-20 (1990).
2. Vistoli, G. et al. Advanced glycoxidation and lipoxidation end products (AGEs and ALEs): an overview of their mechanisms of formation. *Free Radic. Res.* **47**, 3-27 (2013).
3. Ciulla, M. Fibrosis, enzymatic and non-enzymatic cross-links in hypertensive heart disease. *Cardiovasc. Hematol. Disord. Drug Targets* **11**, 61-73 (2011).
4. Zamora, R., Alaiz, M. & Hidalgo, F. J. Determination of ε-N-pyrrolylnorleucine in fresh food products. *J. Agr. Food Chem.* **47**, 1942-1947 (1999).
5. Zamora, R., Alaiz, M. & Hidalgo, F. J. Contribution of pyrrole formation and polymerization to the nonenzymatic browning produced by amino− carbonyl reactions. *J. Agr. Food Chem.* **48**, 3152-3158 (2000).
6. Glenn, J. V. et al. Confocal Raman microscopy can quantify advanced glycation end product (AGE) modifications in Bruch's membrane leading to accurate, nondestructive prediction of ocular aging. *Faseb J.* **21**, 3542-3552 (2007).

7. Czamara, K. et al. Raman spectroscopy of lipids: a review. *J. Raman Spectrosc.* **46**, 4-20 (2015).

8. Miura, T. et al. Raman spectroscopic study on the copper (II) binding mode of prion octapeptide and its pH dependence. *Biochemistry* **38**, 11560-11569 (1999).

9. Ramasamy, R. Vibrational spectroscopic studies of imidazole. *Arm. J. Phys.* **8**, 51-55 (2015).

10. Tuma, R. Raman spectroscopy of proteins: from peptides to large assemblies. *J. Raman Spectrosc.* **36**, 307-319 (2005).

11. Movasaghi, Z., Rehman, S. & Rehman I. Raman spectroscopy of biological tissues. *Appl. Spectrosc. Rev.* **42**, 493-541 (2007). DOI:10.1080/05704920701551530.

12. Lambert, J. B. et al. *Introduction to organic spectroscopy* (Macmillan, New York, 1987:).

13. Bindi, L. et al. In *Highlights in mineralogical crystallography*. (Walter de Gruyter GmbH, New York, 2016).

14. Downs, R. T. *The RRUFF Project: an integrated study of the chemistry, crystallography, Raman and infrared spectroscopy of minerals.* (Program and Abstracts of the 19th General Meeting of the International Mineralogical Association, Kobe, 2006).

15. Pokines, J. T. & Symes, S. A. *Manual of forensic taphonomy* (CRC Press, Boca Raton, 2013).

16. Fernandez-Jalvo, Y. & P. Andrews *Atlas of taphonomic identifications: 1001+ images of fossil and recent mammal bone modification*. (Springer, Dodrecht, 2016).

17. Corrado, G. et al. Surface-enhanced Raman and fluorescence joint analysis of soil humic acids. *Anal. Chim. Acta* **616**, 69-77 (2008).

18. Kaye, T. G., Gaugler, G. & Sawlowicz, Z. Dinosaurian soft tissues interpreted as bacterial biofilms. *PLoS One* **3**, e2808 (2008).

19. Ivleva, N. P. et al. Raman microscopy and surface‐enhanced Raman scattering (SERS) for in situ analysis of biofilms. *J. Biophotonics* **3**, 548-556 (1999).

20. Sandt, C. et al. Confocal Raman microspectroscopy as a tool for studying the chemical heterogeneities of biofilms in situ. *J. Appl. Microbiol.* **103**, 1808-1820 (2007).

21. Gupta, N. S. et al. Molecular structure of organic components in cephalopods: Evidence for oxidative cross linking in fossil marine invertebrates. *Org. Geochem.* **39**, 1405-1414 (2008).

22. Evershed, R. P. et al. Volatile compounds in archaeological plant remains and the Maillard reaction during decay of organic matter. *Science* **278**, 432-433 (1997).

23. Ocampo, R. et al. Porphyrins from Messel oil shale (Eocene, Germany): structure elucidation, geochemical and biological significance, and distribution as a function of depth. *Geochim. Cosmochim. Acta*. **56**, 745-761 (1992).

24. Bakel, A. J. & Philp, R. P. The distribution and quantitation of organonitrogen compounds in crude oils and rock pyrolysates. *Org. Geochem*. **16**, 353-367 (1990).

25. Bennett, R. & Czechowski, F. Gallium porphyrins in bituminous coal. *Nature* **283**, 465-467 (1980).

26. Colleary, C. et al. Chemical, experimental, and morphological evidence for diagenetically altered melanin in exceptionally preserved fossils. *Proc. Natl. Acad. Sci.* **112**, 12592-12597 (2015).

27. Lindgren, J. et al. Skin pigmentation provides evidence of convergent melanism in extinct marine reptiles. *Nature* **506**, 484-488 (2014).
28. Woodburne, M. O. & Robinson, P. T. A new late Hemingfordian mammal fauna from the John Day Formation, Oregon, and its stratigraphic implications. *J. Paleont.* **51**, 750-757 (1977).
29. Oser, S. E. & Jackson, F. D. Sediment and eggshell interactions: using abrasion to assess transport in fossil eggshell accumulations. *Hist. Biol.* **26**, 165-172 (2014).
30. Dalman, S. Osteology of a large allosauroid theropod from the Upper Jurassic (Tithonian) Morrison Formation of Colorado, USA. *Vol. Juras.* **12**, 181-196 (2014).
31. Dodson, P. et al. Taphonomy and paleoecology of the dinosaur beds of the Jurassic Morrison Formation. *Paleobiology* **6**, 208-232 (1980).
32. Martin, T., Averianov, A. O. & Pfretzschner, H. U. Mammals from the Late Jurassic Qigu Formation in the southern Junggar Basin, Xinjiang, Northwest China. *Paleobiodivers. Paleoenviron.* **90**, 295-319 (2010).
33. Averianov, A. O. et al. Middle Jurassic vertebrate assemblage of Berezovsk coal mine in western Siberia (Russia). *Global Geology* **19**, 187-204 (2016).
34. Hagdorn, H., Schoch, R. & Schweigert, G. *Der Lettenkeuper - Ein Fenster in die Zeit vor den Dinosauriern* (Palaeodiversity Sonderband, Stuttgart, 2015).
35. Oser, S. E. & Jackson, F. D. Sediment and eggshell interactions: using abrasion to assess transport in fossil eggshell accumulations. *Historical Biology* **26**, 165-172 (2014).
36. Cope, E. D. Discovery of a gigantic dinosaur in the Cretaceous of New Jersey. *Proc. Acad. Nat. Sci. Philadelphia* **18**, 275-279 (1866).

37. Schnyder, J. et al. An Early Cretaceous lacustrine record: organic matter and organic carbon isotopes at Bernissart (Mons Basin, Belgium). *Palaeogeogr. Palaeoclimatol. Palaeoecol.* **281**, 79-91 (2009).

38. Kirkland, J. I. et al. Ankylosaur (Dinosauria) specimens from the Upper Jurassic Morrison Formation. *Mod. Geol.* **23**, 145-177 (1998).

39. Balanoff, A. M., Bever, G. S. & Ikejiri, T. The braincase of *Apatosaurus* (Dinosauria: Sauropoda) based on computed tomography of a new specimen with comments on variation and evolution in sauropod neuroanatomy. *Am. Mus. Novit*. **3677**, 1-32 (2010).

40. Siber, H. J. & Möckli, U. *The stegosaurs of the Sauriermuseum Aathal* (Sauriermuseum Aathal, Aahtal, 2009).

41. Röhl, H. J. et al. The Posidonia Shale (Lower Toarcian) of SW-Germany: an oxygen-depleted ecosystem controlled by sea level and palaeoclimate. *Palaeogeogr. Palaeoclimatol. Palaeoecol.* **165**, 27-52 (2001).

42. Sauer, E. F. Taxonomic evidence and evolutionary interpretation of *Psammornis*. *Bonn. Zool. Beitr.* **20**, 290-310 (1969).

43. Liang, X. et al. Dinosaur eggs and dinosaur egg-bearing deposits (Upper Cretaceous) of Henan Province, China: occurrences, palaeoenvironments, taphonomy and preservation. *Prog. Nat. Sci.* **19**, 1587-1601 (2009).

44. Grellet-Tinner, G. & Fiorelli, L. E. A new Argentinean nesting site showing neosauropod dinosaur reproduction in a Cretaceous hydrothermal environment. *Nat. Commun*. **1**, 32 (2010).
